# Acupuncture treatment for Sjögren’s syndrome: a narrative review on mechanism of treatment and clinical application

**DOI:** 10.3389/fimmu.2026.1831496

**Published:** 2026-08-14

**Authors:** Xinyi Pu, Yihua Fan, Yongfang Yang, Zinan Guo, Bing Fan, Di Zhang

**Affiliations:** 1 Department of Rheumatism and Immunity, Hospital of Chengdu University of Traditional Chinese Medicine, Chengdu, Sichuan, China; 2 School of Clinical Medicine, Chengdu University of Traditional Chinese Medicine, Chengdu, Sichuan, China; 3 School of Pharmacy, Chengdu University of Traditional Chinese Medicine, Chengdu, Sichuan, China; 4 Department of Rheumatology and Immunology, Affiliated Hospital of Shandong University of Traditional Chinese Medicine, Jinan, Shandong, China; 5 College of Traditional Chinese Medicine, Shandong University of Traditional Chinese Medicine, Jinan, Shandong, China

**Keywords:** acupuncture, complementary therapies, controlled clinical trial, mechanism, Sjögren's syndrome

## Abstract

Sjögren’s syndrome (SS) is a systemic autoimmune disorder whose precise pathogenic mechanisms remain to be fully elucidated. Although Western medicine treatment has definite efficacy, it has disadvantages such as long medication cycles and many adverse reactions. According to literature review, traditional Chinese therapy acupuncture helps regulate immune disorders in SS patients. This regulation effectively alleviates symptoms such as xerostomia, xerophthalmia, fatigue, and joint pain, while also improving emotional disturbances like anxiety and depression. Nevertheless, most available relevant clinical studies are flawed by single-center design, small sample sizes and insufficient blinding, resulting in poor overall methodological quality and limited credibility of the clinical evidence. Furthermore, one randomized controlled study found that acupuncture yielded no superior effects on core symptom relief relative to sham acupuncture, with the between-group difference failing to reach statistical significance. This narrative review comprehensively synthesizes the available evidence, sorts out the potential mechanisms underlying acupuncture for Sjögren’s syndrome — including regulating pro-inflammatory factors such as TNF-α and IL-17, modulating immunoglobulin levels, and restoring Th17/Treg balance — and summarizes relevant clinical studies. Meanwhile, it identifies common limitations of current research, namely insufficient sample sizes, non-standard trial protocol design, and poor methodological rigor. This review aims to provide references for future clinical trials, as well as novel insights and strategic directions for the clinical application of acupuncture in the treatment of Sjögren’s syndrome.

## 1 Introduction

Sjögren’s syndrome (SS), a chronic autoimmune disorder affecting the exocrine glands, presents with lymphocytic infiltration of the involved glands. Symptoms of xerostomia and xerophthalmia result from the involvement of the salivary and lacrimal glands (1). The dysfunction of exocrine glands, including the lacrimal and salivary glands, primarily leads to ocular and oral dryness, which defines SS. As the disease progresses, extra-glandular organs may become involved, eventually developing into a systemic disorder affecting multiple organs and systems. The classification of SS includes primary and secondary subtypes (2). Without concurrent connective tissue diseases, primary Sjögren’s syndrome (pSS) occurs. Secondary Sjögren’s syndrome, however, comes into being based on a confirmed diagnosis of other connective tissue diseases (e.g., rheumatoid arthritis, systemic lupus erythematosus, dermatomyositis), along with symptoms such as xerostomia and xerophthalmia. According to epidemiological studies, SS has a prevalence of 0.3%–0.7% in China (3), with the gender ratio being 1 male to 20 females (4). Current treatment for SS primarily aims to relieve symptoms and protect affected glands. Although topical agents like cyclosporine, artificial tears, pilocarpine, and cevimeline can ease dryness in SS patients, their moisturizing effects are merely temporary (5). Owing to the absence of long-lasting efficacy, frequent dosing is necessary, which inevitably compromises patients’ quality of life. In randomized controlled trials (RCTs), systematic reviews, and meta-analyses, biologics (e.g., telitacicept) (6) and immunosuppressants (e.g., hydroxychloroquine sulfate) (7) have failed to show obvious clinical benefits. Additionally, these agents are associated with issues such as unstable efficacy, high drug prices, adverse drug reactions (e.g., increased infection risk, hepatotoxicity, nephrotoxicity), and poor tolerability (8, 9). Immune dysregulation is the fundamental cause of SS, and traditional Chinese medicine (TCM) can regulate immune cell balance and restore immune homeostasis (4, 10). As a traditional non-pharmacological intervention in TCM, acupuncture is a safe and effective therapeutic approach with prominent advantages in treating SS (11). This paper reviews the mechanisms and current clinical status of acupuncture in the management of SS in recent years

## 2 Mechanisms of Sjögren’s syndrome

SS is a multifactorial disease whose pathogenesis remains incompletely understood. It is closely associated with immune homeostasis dysregulation (12, 13), genetic susceptibility (14, 15), viral infections (16), and intestinal microenvironment (17, 18). According to the generally acknowledged hypothesis of SS pathogenesis, in genetically susceptible hosts, environmental triggers (such as infection, radiation, and trauma) may damage salivary gland epithelial cells (SGECs), leading to the activation of quiescent epithelial cells and the subsequent dysregulation of the immune system (19, 20). Local autoimmune responses are sustained through the interplay of epithelial cells, innate immunity, and adaptive immunity during this process (20). The most typical pathological finding in SS biopsy specimens is focal lymphocytic sialadenitis (FLS), which exhibits a sensitivity and specificity of over 80% (21). FLS is defined as aggregates of ≥50 mononuclear cells (predominantly lymphocytes) surrounding ducts or blood vessels (22). A correlation between lymphocyte subsets and inflammation severity has been documented in studies: T cells are the predominant cells in mild lesions, which are mainly linked to periepithelial changes, whereas B cells characterize more severe and widespread lesions, accompanied by immune complex-mediated features (23). Importantly, autoreactive effector T cells and memory T cells serve as key mediators in pathogenesis, causing direct tissue damage and enhancing the overactivation of antibody-producing B cells, which ultimately leads to systemic manifestations in pSS (24). A variety of cytokines, including interferon-γ (IFN-γ), interleukin-7 (IL-7), and IL-21, are secreted by infiltrating immune cells in SS (25–27). Via activation of the JAK/STAT1 pathway, IFN-γ induces ferroptosis in SGECs from SS patients, which compromises salivary gland function (28). In SS, IL-7 is capable of activating IFN-γ and facilitating interactions between T cells and SGECs (29), whereas IL-21 promotes B cell proliferation, terminal differentiation into plasma cells, and immunoglobulin class switching (30–32). Therefore, proinflammatory cytokines act as a link connecting immune dysregulation and functional impairment in SS (33).It is generally recognized that gut microbiota—particularly bacterial communities—are essential for generating both innate and adaptive immune responses (34). The human immune system relies heavily on gut microbiota for its development, while the microbiota also provides defense against the excessive proliferation of pathogenic microorganisms (35). Meanwhile, research has shown a correlation between the dynamics of human immune cells and the gut microbiome, which indicates that the gut microbiome is responsible for regulating the immune system (36). Gut dysbiosis, defined as an imbalanced state of the gut microbiota, impairs the intestinal mucosal barrier through host-microbe interactions. This leads to dysregulation of intestinal mucosal immunity and increased production of pro-inflammatory cytokines such as IL-1, IL-6, IL-17, and TNF-α, which subsequently induce chronic inflammatory diseases (37, 38). Several studies, in fact, have indicated that patients suffering from Sjögren’s syndrome exhibit marked gut dysbiosis (17, 39–42). At the same time, a study demonstrated that pSS patients’ gut microbiota is characterized by lower diversity and richness, fewer beneficial or commensal butyrate-producing bacteria, as well as a higher ratio of opportunistic pathogens that possess pro-inflammatory activity. This may compromise intestinal barrier function, thereby facilitating pSS-related inflammatory processes by enhancing pro-inflammatory cytokine production, decreasing the release of the anti-inflammatory cytokine IL-10, and reducing peripheral FOXP3 mRNA expression (43).

The investigation of genetic determinants in primary Sjögren’s syndrome (pSS) is still relatively preliminary. However, recent studies have begun to identify the familial aggregation of this disease, pinpoint specific high-risk alleles, and even classify affected individuals based on their global gene expression profiles (44). As an illustration, a meta-analysis revealed a notable positive association between the IRF5 gene’s CGGGG insertion/deletion polymorphism and the development of pSS, which suggests that this polymorphism could function as a potential risk factor for pSS (45). In parallel, investigations utilizing weighted gene co-expression network analysis have effectively detected critical genes and signaling pathways linked to Sjögren’s syndrome in both patients and healthy controls, among which EIF2AK2, GBP1, PARP12, and PARP14 function as primary hub genes (46). At present, the remaining challenges are to clarify the biological functions of specific risk alleles and figure out how to apply these data to assist in the early diagnosis of the disease as well as the therapeutic stratification of patients (44).

SS is a chronic autoimmune inflammatory disease (47). The core pathological manifestations include excessive immune response and persistent chronic inflammation, which eventually lead to irreversible damage to exocrine glands. 30% to 70% of patients will experience multi-system involvement throughout the course of the disease (48–50). To date, the medical community has not found a specific and effective therapy that can cure primary Sjögren’s syndrome and completely alleviate the stubborn fatigue, dryness of the entire mucous membranes, and systemic pain in the majority of patients. Moreover, the treatment cost of this disease is high, and the satisfaction of both doctors and patients with the existing therapies is generally low (51). Studies have shown that acupuncture has a regulatory effect on the “neuro-endocrine-immune system network” (51), which can inhibit pathological immune activation, alleviate inflammatory damage to glands, and improve the clinical symptoms of Sjögren’s syndrome (52). The following text will further discuss and analyze the possible role of acupuncture in the treatment of Sjögren’s syndrome.

## 3 Mechanistic studies of acupuncture in treating Sjögren’s syndrome

To systematically clarify the mechanisms underlying acupuncture therapy for pSS, this paper integrates all relevant research evidence in this field and grades the evidence in accordance with unified criteria (see **Table 1** for evidence classification). As shown in **Table 1**, the mechanisms described in Sections 2.1, 2.2 and 2.3 — improvement of inflammatory markers, regulation of immunoglobulins, and modulation of the Th17/Treg balance — are supported by clinical trial data from patients with pSS. In contrast, the mechanisms covered in Sections 2.4 and 2.5, including the regulation of innate immune signaling pathways and immune modulation mediated by the gut-brain axis and intestinal flora, are currently backed mainly by theoretical hypotheses and indirect evidence such as animal studies from other disease areas, corresponding to a relatively low level of evidence. Their complete regulatory networks and core molecular targets remain to be verified by further in-depth research.

**Table 1:** Mechanistic research on the acupuncture trial for SS.

Research content	Direct clinical evidence	Indirect animal evidence	Indirect extrapolation evidence	Theoretical evidence
Acupuncture regulates the cholinergic anti-inflammatory pathway	(54, 56, 64, 76)	(52, 55, 57, 58, 61, 62, 76)	(63, 66–69, 74, 75)	(53, 59, 60, 65, 70–72)
Acupuncture modulates serum immunoglobulin levels	(78, 80, 81, 84, 87)	/	(83)	(77, 79, 82, 85, 86)
Acupuncture regulates Th17/Treg balance	(94, 96, 98, 103)	(97)	(99–102)	(89–93, 95)
Acupuncture regulates the innate immune response	/	(122)	(110, 113, 120, 121, 123)	(104–109, 111, 112, 114–119)
Acupuncture regulates intestinal flora	/	/	(124–127, 131)	(128–130)

### 3.1 Acupuncture regulates pro-inflammatory factors through the cholinergic anti-inflammatory pathway

In diseases caused by dysregulated autoimmunity, proinflammatory cytokines play a vital role in disease initiation and progression, mediating multi-level interactions among cells, immune factors, and biochemical messengers (53). The abnormally high expression of interleukin-17 (IL-17) and tumor necrosis factor-α (TNF-α) is a characteristic feature of SS (54–56) and plays a significant role in disease progression (57–61). In animal studies, increased expression of IL-17 receptor signaling pathway-associated genes (such as CCL11, CCL7, Fos, Jun, Lcn2) in mice has been shown to promote SS progression, with IL-17 further inducing inflammation-mediated cell death in salivary gland cells (62). TNF-α is a key mediator of inflammatory responses, and its elevated expression is frequently associated with inflammatory diseases leading to salivary gland hypofunction (54). Studies have shown that TNF-α promotes the synthesis of matrix metalloproteinase 2 in acinar cells, potentially disrupting acinar cell structure (63, 64).

Studies have demonstrated that efferent vagus nerves can suppress the release of pro-inflammatory cytokines and modulate inflammation in real time (65–69). Acetylcholine (ACh) released via vagal efferent fibers regulates immune responses in human macrophages through α7 nicotinic acetylcholine receptors (α7nAchRs), inhibits NF-κB, and thereby blocks the synthesis and secretion of cytokines such as TNF-α and IL-1 (65, 66, 70–72). Research has shown that IL-17 is primarily secreted by CD4+ T cells, and TLR2 ligation induces IL-17 production via the NF-κB pathway (73). One study reported that acupuncture alleviates inflammatory responses in mouse models of xerophthalmia by upregulating α7 nicotinic acetylcholine receptors (α7nAchRs) and downregulating NF-κB expression, which in turn reduces pro-inflammatory factors including TNF-α (52). Another studies indicated that acupuncture suppresses NF-κB expression and downregulates IL-4, IL-17 (74), TNF-α (75) and other mediators in rat models to exert anti-inflammatory effects. Regarding IL-17 and TNF-α, research demonstrates that acupuncture is capable of reducing serum concentrations of IL-17 and TNF-α in patients, suppressing lymphocytic infiltration in the submandibular glands of mice, regulating IL-17 and TNF-α expression effectively, and alleviating salivary gland inflammation (76). Based on the above relevant studies, the cholinergic anti-inflammatory pathway mediated by α7nAChR may serve as a potential pathway underlying the efficacy of acupuncture. Nevertheless, current evidence merely involves simple analyses of related pro-inflammatory factors, and direct data supporting this specific pathway are still lacking.

### 3.2 Acupuncture modulates serum immunoglobulin levels

Immunoglobulins (Ig) are proteins dependent on B cell activation and maturation, involved in antigen presentation and recognition, as well as the activation of other immune cells (primarily T lymphocytes) (77). Studies have shown that salivary gland lymphocytes in SS patients synthesize significantly increased amounts of IgG, IgM, and IgA (78). Clinically, IgG is widely recognized as a marker of disease activity in SS (79), and a weak correlation has been reported between increased fatigue and elevated serum IgG and IL-17 levels (80). A 10-year follow-up study revealed an association between extra-glandular involvement and elevated IgG titers, with 75% of patients presenting with hypergammaglobulinemia (81). Currently, some scholars (82) hold the view that acupuncture exerts a bidirectional regulatory effect on the immune system. It can boost immune function for the treatment of immunosuppressive disorders; by contrast, it produces immunosuppressive effects in autoimmune diseases so as to maintain immune homeostasis. A randomized controlled trial (83) demonstrated that acupuncture treatment can effectively improve immune function in patients with sepsis, with a more prominent elevation of IgG levels observed in the acupuncture group compared with other groups. An RCT has shown that acupuncture can effectively reduce serum IgG levels in patients with Sjögren’s syndrome (SS) (84). This effect may be attributed to acupuncture-induced activation of neural networks, which modulate the immune system and promote the secretion of multiple bioactive substances via peripheral, spinal and supraspinal pathways (85, 86). Two additional studies demonstrated that compared with the control group, patients in the acupuncture group achieved marked improvements in serum IgG levels and systemic symptoms (87, 88), and such changes in IgG were consistent with the findings of the aforementioned studies. Overall, existing clinical studies have only preliminarily verified that acupuncture can ameliorate serum IgG levels and systemic manifestations in patients with SS. To date, researchers have not completely clarified the distinct neuroimmunological mechanisms behind the correlation of these two outcomes. Further fundamental studies focused on Sjögren’s syndrome cohorts are therefore needed to confirm their internal regulatory routes and valid intervention targets.

### 3.3 Acupuncture can regulate the imbalance between T helper 17 cells and regulatory T cells

As a chronic autoimmune disorder, SS is driven by the abnormal activation of T and B lymphocytes. While B cells are responsible for the excessive generation and release of autoantibodies, T lymphocytes represent the dominant infiltrating cell population throughout most pathological stages of SS, and their activation contributes to tissue damage and impaired secretory function (89) Previous studies have characterized various subsets of T helper cells, among which T helper 17 (Th17) cells, regulatory T (Treg) cells, and follicular helper T cells are included (90). As potent promoters of tissue inflammation, Th17 cells are found in the afflicted tissues of various autoimmune disorders and are implicated in the pathogenic mechanisms of rheumatoid arthritis, psoriasis, inflammatory bowel disease, and Sjögren’s syndrome (91). Conversely, Regulatory T (Treg) cells are essential for regulating the complex immune responses required for homeostasis (92, 93), and they play a critical role in both the development and progression of SS (94, 95). Meanwhile, numerous studies have shown that the pathogenesis of SS is associated with an imbalance in the Th17/Treg cell ratio (91, 96–98).

RORγt is a key transcription factor governing the differentiation of Th17 cells (99), whereas Foxp3 functions as a transcriptional repressor that inhibits the activity of RORγt (100, 101). One study (102) demonstrated that electroacupuncture at acupoints ST9 and LR3 in rats downregulated the expression of RORγt protein and upregulated the expression of Foxp3 and TGF-β1. High concentrations of TGF-β1 can induce the differentiation of Treg cells (101). Therefore, electroacupuncture suppresses the generation of Th17 cells while promoting the production of Treg cells, thereby balancing the Th17/Treg cell ratio. A randomized controlled trial confirmed that acupuncture can correct immune dysregulation by regulating the Th17/Treg ratio and reduce inflammatory markers in patients with primary Sjögren’s syndrome (pSS), including IgG, ESR and CRP (103). Such immunomodulation not only enhanced exocrine gland performance but also markedly alleviated the hallmark symptoms of xerostomia and xerophthalmia. In summary, the regulation of Th17/Treg cell balance by acupuncture may be an important potential mechanism underlying its immunomodulatory effects. This has been preliminarily confirmed by clinical studies; however, current evidence regarding regulation at the transcription factor level is mainly derived from animal experiments, and its specific action pattern in human patients with pSS has not been directly verified.

### 3.4 Acupuncture regulates the innate immune response

Innate immune responses not only participate crucially in the initial stages of the pathogenesis of SS but also contribute to the progression of chronic inflammation (104). Innate immunity performs a more vital and fundamental role in host defense (105). Primary cells involved in innate immune responses include dendritic cells, natural killer (NK) cells, epithelial cells and macrophages (106–108). Innate immunity is initiated via pattern recognition receptors (PRRs), which recognize pathogen-associated molecular patterns (PAMPs) derived from exogenous microorganisms (106, 109). Toll-like receptors (TLRs), the first identified class of PRRs, are capable of recognizing 10 distinct types of human PAMPs (109). In patients with SS, TLR2 is expressed in salivary gland tissue, and this expression correlates with the severity of salivary gland inflammation (73). Moreover, stimulation with TLR2 ligands in cultured salivary gland epithelial cells (SGECs) isolated from SS patients promotes the secretion of IL-15 (110), which is involved in activating the proliferation of T and B cells as well as sustaining natural killer cells (111, 112).

A prominent hallmark of patients with SS is the aberrant deposition of mucins MUC5B and MUC7 within the extracellular matrix of salivary glands. Further investigations have revealed that these mucins can be recognized by epithelial TLR4, which markedly elevates the levels of CXCL8, TNF-α, IFN-α, IFN-β, IL-6 and IL-1β, thereby driving the development of chronic inflammatory status in SS (113).

Dendritic cells (DCs) are specialized antigen-presenting cells (APCs) (114). They recognize microorganisms, secrete pro-inflammatory cytokines, undergo maturation and acquire the capacity to activate T cells, with TLRs delivering critical signals throughout these processes (115, 116). TLR signaling induces the upregulation of co-stimulatory molecules, which are indispensable for T cell proliferation. Macrophages eliminate apoptotic and necrotic cells through phagocytosis. In addition, they internalize and process invading pathogens via phagocytosis, resulting in pathogen clearance, or present antigenic peptides on the cell surface through MHC class I and II molecules to trigger adaptive immune responses (117). TLRs mediate multiple macrophage functions, including phagocytosis (118, 119), antigen processing and presentation (120), and the initiation of adaptive immune responses (115, 121).

Taken together, TLRs exert a central function in innate immune responses. Recently, a study (122) has shown that electroacupuncture can reduce the expression levels of TLR2 and TLR4 in the corneal and lacrimal gland tissues of mice by inhibiting the HMGB1-related signaling pathway, significantly increase tear flow, and reduce corneal staining and corneal stromal inflammation. Another study (123) reported that pre-treatment with electroacupuncture in endotoxemic rats inhibits Ca²^+^ influx and blocks the TLR4-NF-κB signaling pathway, substantially suppressing the production of LPS-induced pro-inflammatory cytokines including TNF-α, IL-1β and IL-6. The above findings provide certain preclinical experimental evidence that acupuncture alleviates inflammation of exocrine glands and improves glandular secretory function via regulating innate immune responses. Nevertheless, it should be clarified that both studies were conducted on animal models. Since endotoxemic rat models feature pathological conditions distinct from the autoimmune pathogenesis underlying pSS, the modulatory function of this pathway lacks direct verification in human pSS patients and merely offers theoretical clues to interpret acupuncture’s putative therapeutic mechanisms.

### 3.5 Acupuncture regulates the intestinal flora:a missing research component

As mentioned above, dysbiosis of the intestinal flora is closely linked to the onset and progression of SS. Multiple studies have indicated that acupuncture can remodel the intestinal microbiota toward a more beneficial profile: it elevates the abundance of beneficial bacteria and reduces the quantity of pathogenic bacteria (124–127). Meanwhile, acupuncture decreases the levels of intestinal fatty acid-binding protein, D-lactate, LPS and lipopolysaccharide-binding protein, and lowers the peripheral blood neutrophil count, thereby restraining systemic inflammatory responses secondary to inflammation (124).

**Figure 1:**
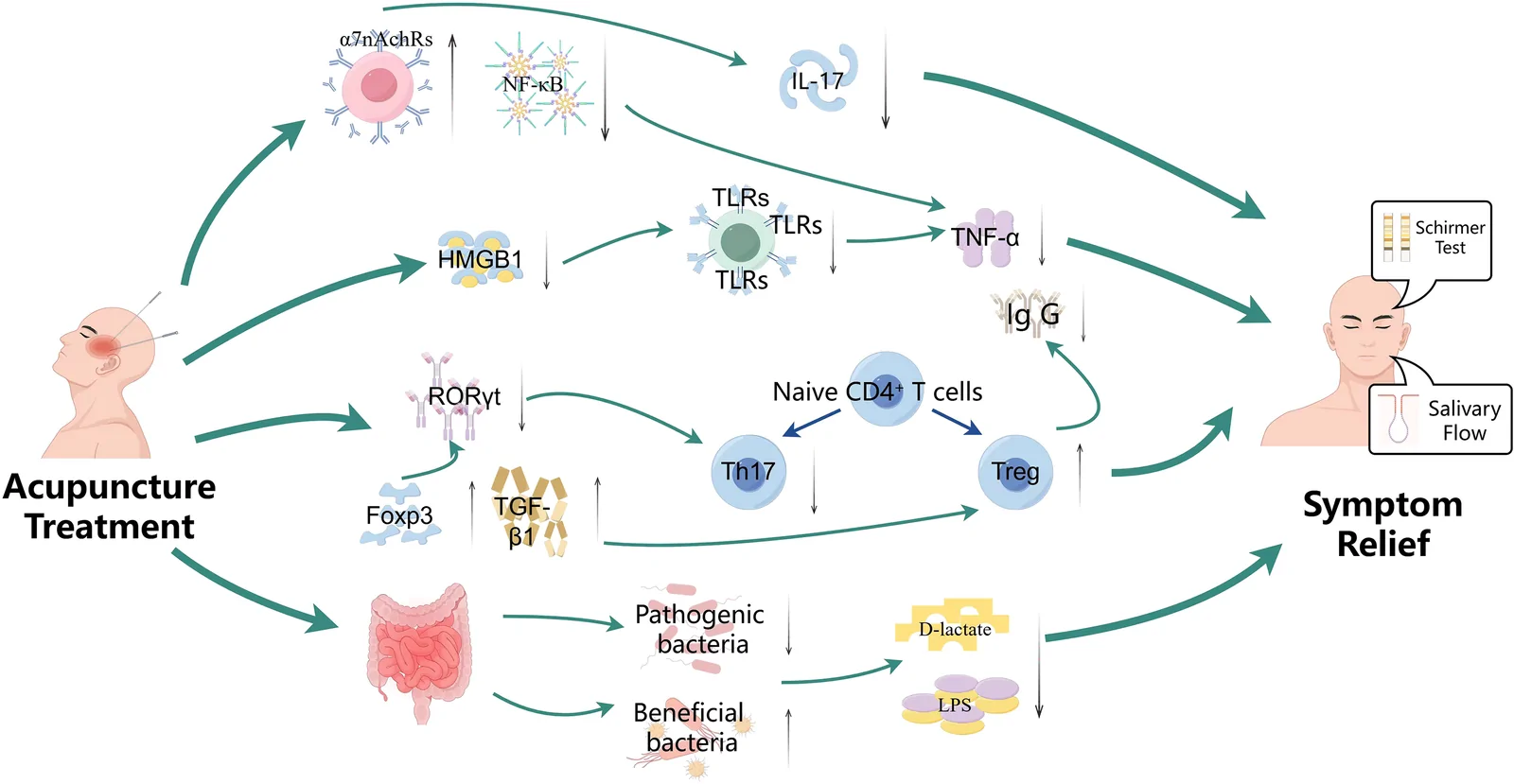
Mechanism of acupuncture treatment for Sjögren’s syndrome. Created by Figdraw (www.figdraw.com). Acupuncture therapy can upregulate α7nAchRs and downregulate NF-κB, thereby reducing the production of pro-inflammatory factors including IL-17 and TNF-α. It can also suppress HMGB1 to decrease the expression of TLRs and further lower TNF-α secretion. Additionally, acupuncture reduces the expression of RORγt while elevating Foxp3 and TGF-β1, so as to correct the Th17/Treg imbalance and inhibit IgG overproduction. Moreover, acupuncture modulates intestinal flora by enriching beneficial bacteria and suppressing pathogenic bacteria, accompanied by reduced levels of lipopolysaccharide and D-lactate. Collectively, acupuncture alleviates patients’ symptoms through these multi-pathway mechanisms.

Nevertheless, existing research on acupuncture regulating the gut-brain axis mainly focuses on digestive and neurological disorders, such as irritable bowel syndrome (128–130) and mild cognitive impairment (131). Systematic investigations regarding the effects of acupuncture on the intestinal microbiota in SS patients and the mechanistic pathways underlying acupuncture treatment for Sjögren’s syndrome via the gut-brain axis remain scarce, representing a key research direction to be addressed in future studies. There is a lack of randomized controlled trials focusing on patients with primary Sjögren’s syndrome that directly confirm acupuncture can reshape the intestinal flora structure of patients. In addition, causal verification of the “gut microbiota–gut-brain axis–gland immunity” axis in SS animal models is absent. The insufficiency of relevant research in this field represents a key research direction to be prioritized in future investigations. The mechanism of acupuncture treatment for Sjögren’s syndrome is shown in the **Figure 1**.

## 4 Search methods

**Figure 2:**
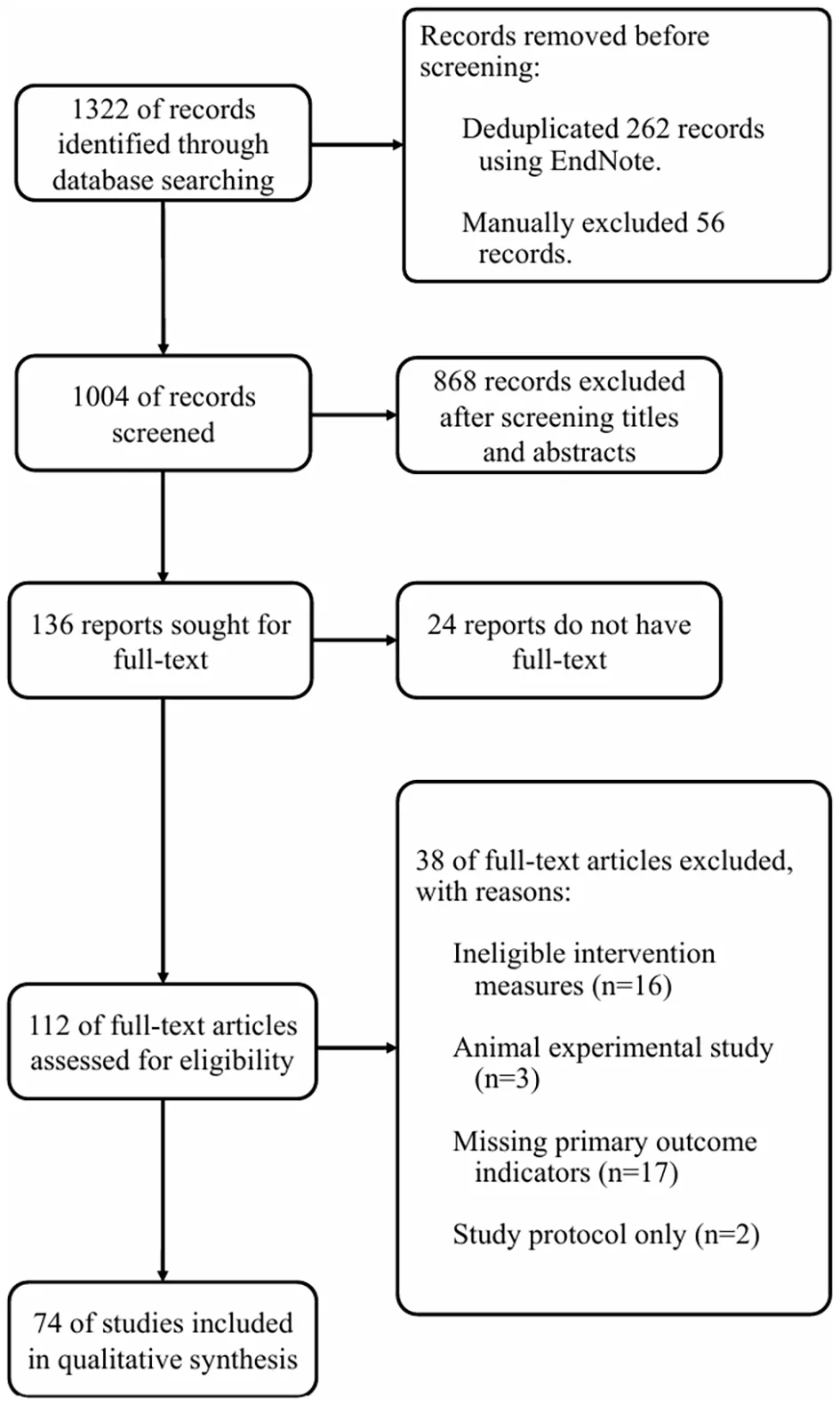
PRISMA flow diagram of literature screening and selection.

This paper is a narrative review. To synthesize evidence rigorously in the section discussing the clinical application of acupuncture for Sjögren’s syndrome (SS), literature retrieval for this part shall be performed in accordance with the PRISMA guidelines. The detailed search flow is illustrated in **Figure 2**.

### 4.1 Search strategy

Foreign electronic databases were searched, including the Cochrane Library, Web of Science, PubMed, and Embase. Chinese databases included China National Knowledge Infrastructure (CNKI), Chinese Biomedical Literature Database (CBM), Wanfang Data, and VIP Chinese Journal Database. The following keywords were adopted for retrieval across all the above databases: (((((((((Acupuncture Treatment) OR (Acupuncture Treatments)) OR (Treatment, Acupuncture)) OR (Therapy, Acupuncture)) OR (Pharmacoacupuncture Treatment)) OR (Treatment, Pharmacoacupuncture)) OR (Pharmacoacupuncture Therapy)) OR (Therapy, Pharmacoacupuncture)) OR (Acupotomy)) OR (Acupotomies);(“Sjogren’s Syndrome”[Mesh]) OR (((((Sjogrens Syndrome) OR (Syndrome, Sjogren’s)) OR (Sjogren Syndrome)) OR (Sicca Syndrome)) OR (Syndrome, Sicca)).Complete PubMed search strategies can be found in **Supplementary Table 1**.

### 4.2 Inclusion criteria

Studies eligible for inclusion were randomized controlled trials, cross-sectional studies, retrospective studies, recent high-quality systematic reviews with or without meta-analyses, and non-peer-reviewed dissertations that adopted acupuncture therapy (covering all acupoint stimulation modalities, including filiform needle acupuncture, electroacupuncture, fire needle, auricular point pressing, and pricking cupping) for the treatment of Sjögren’s syndrome. In addition, basic research that helps further elucidate the mechanism of acupuncture against Sjögren’s syndrome was also included.

### 4.3 Exclusion criteria

Articles focusing on qigong or massage, conference abstracts, opinion statements, commentaries, experience summaries, case reports, duplicate studies and animal experiments or those merely mentioning “acupuncture” only as institutional affiliation without relevant research content were excluded.

## 5 Results

### 5.1 Practical clinical application of acupuncture in managing Sjögren’s syndrome

Acupuncture, as a traditional non-pharmacological therapy in traditional Chinese medicine (TCM), is widely used in the clinical intervention of SS. A 2025 meta-analysis showed that acupuncture provides better clinical efficacy for Sjögren’s syndrome than conventional treatment methods (132).

#### 5.1.1 Monotherapy with acupuncture

Acupuncture monotherapy is defined as the exclusive use of acupuncture intervention for the management of Sjögren’s syndrome (SS). Several small-scale clinical studies have suggested that this intervention confer certain clinical benefits compared with alternative therapeutic regimens. From the perspective of TCM, the core pathological understanding of Sjögren’s syndrome (SS) centers on yin deficiency, which arises from the consumption of body fluids by dry-heat and subsequently progresses to a pathological state characterized by the intermingling of deficiency, phlegm, and toxins (133, 134). Therefore, acupuncture treatment for SS prioritizes the selection of acupoints aimed at nourishing yin, clearing heat, generating body fluids, and moistening dryness—such as Sanyinjiao (SP 6), Lianquan (CV 23), and Taixi (KI 3).

##### 5.1.1.1 Acupuncture group and blank control group

**Figure 3:**
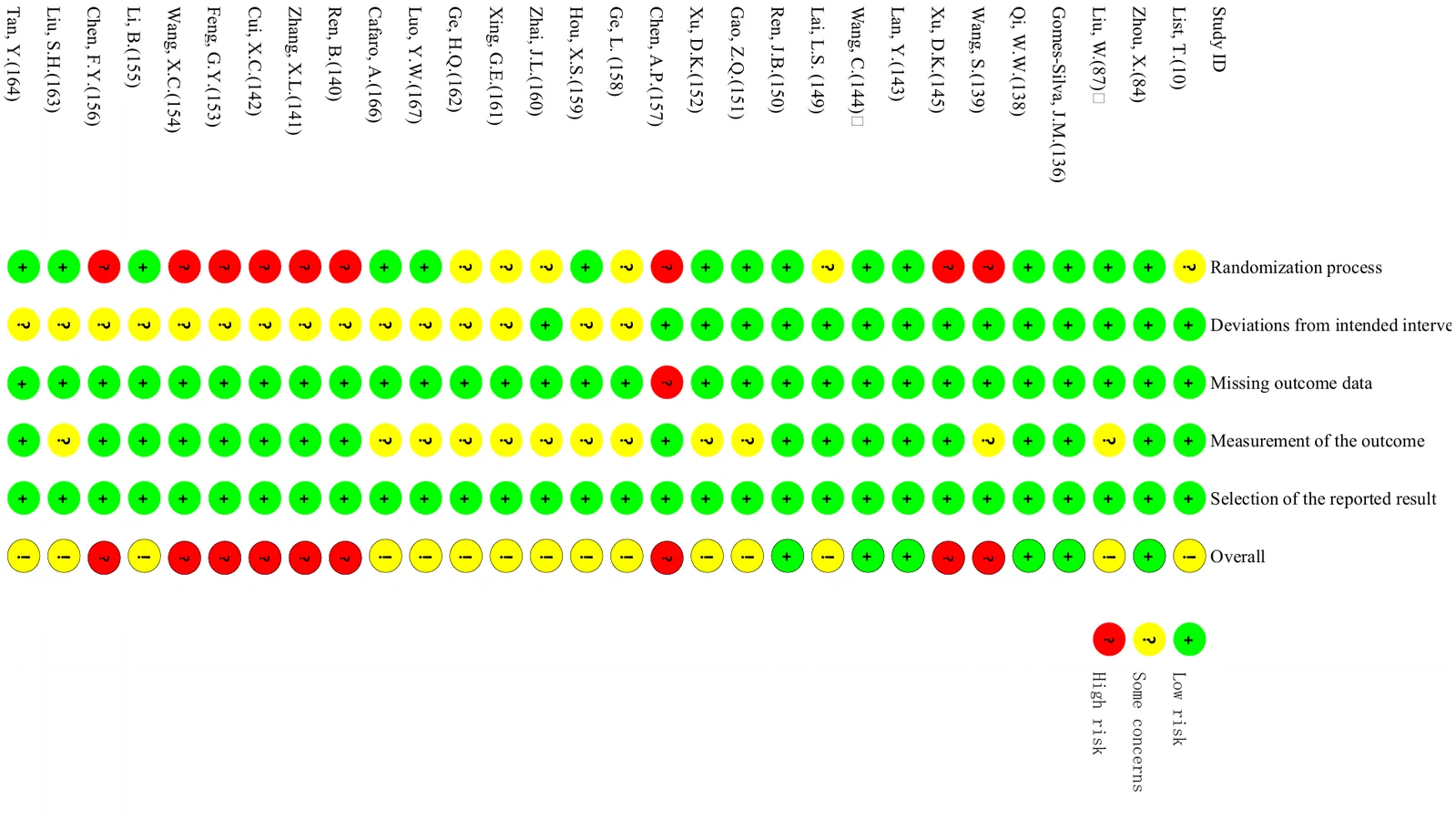
Risk assessment of bias for the included RCT.

Participants in the acupuncture group underwent acupuncture treatment, while those in the blank control group served as a self-control or received sham acupuncture treatment or no treatment at all. A retrospective study (135) on acupuncture for treating xerostomia symptoms in SS patients showed that after a 6-month follow-up, patients who received 24 acupuncture sessions had a significant difference in their tear solution flow rate (Schirmer’s Test Filter Paper Rate, SFR) compared to before receiving acupuncture treatment, and their symptoms of xerostomia and xerophthalmia were markedly relieved. Moreover, patients undergoing additional acupuncture showed a higher median SFR in the long-term (3-year) follow-up than those who discontinued therapy. This suggests that acupuncture treatment not only significantly improves the SFR of SS patients at 6 months, but that subsequent additional acupuncture sessions can also sustain this SFR improvement for as long as 3 years. In this randomized, double-blind, placebo-controlled, single-center, prospective trial (Low risk——The risk assessment was conducted using Cochrane RoB 2, as detailed in **Figure 3**. In subsequent randomized controlled trials, the risk of bias will be described as “high risk”, “Some concerns “ or “low risk”.), 46 SS patients with xerostomia and xerophthalmia were selected and assigned to either the acupuncture group or the sham acupuncture group. Ultimately, 27 patients completed the trial, with 15 receiving acupuncture at points KI 6, ST 6, LI 4, CV 24, TE 23, BL 2 and 12 undergoing sham acupuncture. After the treatment concluded, the acupuncture group exhibited significant improvements in both the total EULAR Sjögren’s Syndrome Patient Reported Index (ESSPRI) score and the ESSPRI dryness score, with these positive effects persisting for at least 4 weeks. Moreover, with the passage of time, the acupuncture group showed improvements in the ESSPRI fatigue domain, ESSPRI pain domain, Oral Health Impact Profile (OHIP-14), Xerostomia Inventory (XI), unstimulated whole salivary flow (UWSF), and Schirmer test (ST) (136). A cross-sectional study also demonstrates a high prevalence of anxiety and depression among patients with primary Sjögren’s syndrome (pSS) (137). Acupuncture treatment can effectively improve psychological distress such as anxiety and depression in patients who were diagnosed with pSS (Low risk) (138). Another earlier controlled trial (Some concerns), which randomized 21 pSS patients to acupuncture or a no-treatment control, found that a notable proportion of participants reported subjective symptomatic improvement, particularly in xerostomia, following the intervention (10). The specific effect size values of the acupuncture group and the control group in this section are shown in **Table 2**.

**Table 2:** The specific effect size values of the acupuncture group and the control group.

Included study	Groups	Sample size	Primary outcome indicators	Effect size	P-value	Study type
List, T. (10)	Acupuncture group/Blank control group	21 (10 in acupuncture group, 11 in blank control group)	①Unstimulated whole saliva flow;②Paraffin-stimulated whole saliva flow; ③ Xerostomia visual analogue scale (VAS)	①Unstimulated whole saliva flow: Acupuncture group: baseline 0.0 (0.0–0.2), post-treatment 0.0 (0.0–0.6); Control group: baseline 0.0 (0.0–0.7), post-treatment 0.0 (0–0.2) ②Paraffin-stimulated whole saliva flow: Acupuncture group: baseline 0.6 (0.0–1.2), post-treatment 1.2 (0.05–2.6); Control group: baseline 0.5 (0.0–2.4), post-treatment 0.6 (0.1–2.5) ③Xerostomia VAS: Acupuncture group: baseline 7.2 (4.5–10.0), post-treatment 5.5 (3.2–10.0); Control group: baseline 6.3 (0.0–9.5), post-treatment 6.8 (0.0–9.5)	①No statistical significance; ②Intra-group P ≤ 0.05 in acupuncture group;③Intra-group P ≤ 0.05 in acupuncture group	Randomized controlled trial (RCT)
Blom, M. (135)	Pre-treatment/Post-treatment	25	① Unstimulated whole saliva flow rate (UWSFR); ② Stimulated whole saliva flow rate (SWSFR); ③ Changes in subjective symptoms	①Post-treatment UWSFR was significantly higher than baseline; ② Post-treatment SWSFR was significantly higher than baseline; ③ Relative risk (RR)=0.64, odds ratio (OR)=0.43	① P < 0.01; ② P < 0.001;③Not calculated	Retrospective study
Gomes-Silva, J.M. (136)	Acupuncture group/Sham acupuncture group	46 (25 in acupuncture group, 21 in sham acupuncture group)*	①Total EULAR Sjögren’s Syndrome Patient Reported Index (ESSPRI) score;②ESSPRI xerostomia subscore;③ UWSFR; ④ Left eye Schirmer’s test (ST)	①Total ESSPRI: Scores decreased significantly from baseline in acupuncture group, while an upward trend was observed in sham acupuncture group ②ESSPRI xerostomia subscore: Significant reduction from baseline in acupuncture group, slight decline in sham acupuncture group ③UWSFR: Acupuncture group: baseline 0.05, post-treatment 0.04, 4-week follow-up 0.10; Sham group: baseline 0.03, post-treatment 0.02, 4-week follow-up 0.03 ④ Left eye Schirmer’s test: Marked elevation in acupuncture group	①P=0.04;②P= 0.0009;③Not calculated;④P= 0.04	Randomized controlled trial (RCT)
Qi, W.W. (138)	Acupuncture group/Sham acupuncture group	41 (20 in acupuncture group, 21 in sham acupuncture group)	①Xerostomia domain of Sjögren’s Syndrome Disease Activity Questionnaire (HADA); ② Ocular dryness domain of Sjögren’s Syndrome Disease Activity Questionnaire (HADD)	①HADA score: Acupuncture group decreased from 11.85 ± 3.42 at baseline to 5.50 ± 2.67 after 8 weeks of treatment; Sham group changed from 12.90 ± 3.06 to 9.76 ± 3.37 ②HADD score: Acupuncture group decreased from 11.80 ± 3.83 at baseline to 4.55 ± 2.80 after 8 weeks of treatment; Sham group changed from 11.19 ± 3.22 to 8.19 ± 4.46	① P < 0.05; ② P < 0.05	Randomized controlled trial (RCT)

##### 5.1.1.2 Acupuncture group and other treatment control group

The acupuncture group, which received verum needling, demonstrated consistently superior efficacy compared to the control group, which was administered alternative treatment modalities. As shown in a representative study (high risk), 40 SS patients were randomly divided into two therapeutic groups: the control group received intravenous Shengmai Injection combined with artificial tear solution, while the acupuncture group was treated with the Runzao Tongluo method. The enhanced therapeutic effect of the Runzao Tongluo acupuncture method in SS (139) was supported by post-treatment analysis after six weeks, which demonstrated a statistically significant disparity in the total effective rate between the acupuncture group (95.0%) and the control group (60.0%) (*P < 0.05*). In a separate study (Some concerns), 120 SS patients who were assigned to an acupuncture group received treatment at acupoints including PC 3, LR 3, SP 10, SP 6, and KI 3, whereas the control group was administered oral prednisone combined with an artificial tear solution. What the final analysis revealed was a statistically significant disparity in the effective rate between the acupuncture (73.3%) and control (56.7%) groups following the 3-month intervention (*P<0.05*). Therefore, acupuncture treatment for SS is effective and safe (87). In one clinical observational trial (high risk), 90 patients were randomized equally into electroacupuncture treatment group and oral Zhibai Dihuang Pills control group. After 4 weeks, total effective rates were 88.9% and 57.8% respectively (140). A trial (high risk) enrolled 60 randomized patients. Controls received Shengmai Injection plus prednisone; the 30-case treatment group adopted Runzao Tongluo acupuncture (zang-fu Back-Shu, distant meridian and local acupoints). Two months later, effective rates reached 83.3% vs 56.7% (*P<0.05*). The treatment group showed statistically significant improvements in Schirmer test, tear film break-up time (BUT), salivary flow rate, IgG, ANA and RF (P<0.01), with better results than controls for all above markers (*P<0.01*) (141). A small controlled trial (high risk) found higher therapeutic efficacy in the electroacupuncture group versus the anethole trithione control group, with a significant intergroup difference (*P<0.05*) (142). In a clinical observation study, this randomized controlled trial (Low risk) employed a parallel-group design for 60 pSS patients. The experimental intervention integrated oral hydroxychloroquine sulfate tablets with the Xinwu acupuncture technique, which was compared against a control intervention of the same medication coupled with shallow acupuncture. After the 8-week treatment period, the experimental group showed a significantly greater improvement in both ESSPRI scores and Schirmer’s Test (ST) results than the control group. The combination of Xinwu acupuncture technique and Hydroxychloroquine sulfate can effectively improve symptoms of xerostomia and Eye dryness in pSS patients (143). Overall, compared with other comprehensive treatment methods, acupuncture has advantages in improving important indicators and symptoms. In the above clinical observation study (143), the Xinwu acupuncture method achieved better therapeutic effects, suggesting that in clinical treatment, choosing the appropriate acupuncture method is also particularly important.

#### 5.1.2 Acupuncture combined with western medicine

In conclusion, the combined treatment of acupuncture and Western medicine produces a synergistic effect, which results in improved therapeutic outcomes. For example, 52 individuals with pSS were randomly divided into two categories (Low risk): one receiving treatment and the other a control. Oral hydroxychloroquine sulfate tablets served as the monotherapy for patients in the control arm. In contrast, those assigned to the treatment group received the same oral regimen combined with acupuncture targeting Zhaohai (KI 6), Fuliu (KI 7), Sanyinjiao (SP 6) and other relevant acupoints. After a 12-week treatment period, the treatment group showed significant improvements in multiple indicators—including ESSPRI scores, tear and salivary flow rates, ESR, CRP, and RF—when compared to the control group (*P<0.05*). Relative to the control group with an overall effective rate of 69.23%, the treatment group reached a rate of 92.31%, and the gap between the two cohorts was statistically meaningful (*P < 0.05*). Compared with using oral Hydroxychloroquine Sulfate alone, the combination of acupuncture and Hydroxychloroquine Sulfate treatment demonstrates a beneficial effect. It can promote the secretion of exocrine glands, including the lacrimal and salivary glands, significantly relieving the symptoms of xerostomia and xerophthalmia. Moreover, this combined treatment shows good anti - inflammatory properties and reduces the immune response (144). In a clinical observation trial involving 60 SS patients (high risk), the control group received oral Total Glucosides of Radix Paeoniae Alba capsules. What constituted the additional intervention for the treatment group was acupuncture at Back-Shu points, alongside the identical oral medication. The evaluation after treatment indicated that, when compared with the control group, the treatment group presented remarkable enhancements (*P < 0.05*) in Schirmer’s test, break - up time (BUT), and salivary flow rate. Furthermore, the overall effective rate exhibited a statistically significant difference, with the treatment group achieving 93.3% and the control group only 53.3% (*P < 0.05*). These results support the conclusion that the integration of Total Glucosides of Radix Paeoniae Alba capsules with Back-Shu point acupuncture constitutes an effective strategy for mitigating xerostomia and xerophthalmia symptoms in affected patients (145). Peripheral neuropathy stands as the most prevalent neurological complication, and it should be noted that neurologic involvement is a frequently occurring extraglandular manifestation of primary Sjögren’s syndrome (pSS) (146, 147). Peripheral neuropathy (PN) in most cases initially presents with sensory symptoms such as tingling, pricking, numbness, tightness, burning and pain (148). In a clinical study of 60 SS patients with SS (Some concerns), the control group received oral Methylprednisolone and Hydroxychloroquine sulfate tablets. What differentiated the observation group was the administration of Huatan Huoxue acupuncture at points including Fenglong (ST40), Yinlingquan (SP9), and Hegu (LI4), in addition to the identical oral medication. At the three-month mark, the improvement in sensory nerve electrophysiology was significantly greater in the observation group than in the control group (*P < 0.05*). Moreover, the observation group’s total efficacy rate of 86.67% was notably higher than the 66.67% recorded for the control group (*P < 0.05*). Hence, combined therapy with phlegm-resolving and blood-activating acupuncture plus conventional Western medicine yields superior efficacy over Western monotherapy for Sjögren’s syndrome complicated with peripheral neuropathy. Noticeable enhancements were noted in the speed, response time, and loudness of sensory nerve transmissions in both the Median and Common fibular nerves (149). In summary, combined acupuncture and Western medicine therapy may yield comparatively better therapeutic effects for SS than Western medicine alone, and appears to be more effective in improving multiple key outcome indicators.

#### 5.1.3 Acupuncture combined with traditional chinese medicine

Acupuncture combined with Chinese herbal medicine (CHM) is a commonly used comprehensive TCM therapy in clinical practice, with the two modalities exerting a synergistic effect. Compared with other treatment approaches, this combined therapy is suitable for intervention throughout the entire course of Sjögren’s syndrome (SS) and is associated with a low incidence of side effects. Based on clinical observation experience, the author believes that the early use of acupuncture and herbs can help control the condition and assist in reducing Inflammation, while long-term regular treatment can alleviate Disease progression and gradually reduce or even replace the dosage of Western medicine. Evidence for the efficacy of combined acupuncture and herbal medicine is currently constrained by a lack of large-scale RCTs, a limitation especially pertinent given that this strategy is inherently tied to treatment based on syndrome differentiation, a cornerstone principle of TCM. In clinical settings, it is the TCM syndrome differentiation of Sjögren’s syndrome (SS) that directs the choice of specific acupuncture points and herbal formulas. The fundamental pathogenesis, as referenced in (83), can be summarized as follows: Yin deficiency constituting the root, dryness-heat manifesting as the branch; concomitant with consumption of qi and body fluids, and obstruction by blood stasis; and further characterized by dampness retention, heat stagnancy, and collateral obstruction by phlegm.

##### 5.1.3.1 Yin-deficiency is root, dryness-heat manifests as superficial

The consumption and deficiency of qi, blood, and yin-fluid (three essential substances in TCM) constitute the fundamental pathogenesis of this disease. Accordingly, it is typically managed with therapeutic strategies focused on enriching yin, clearing heat, moistening dryness, and promoting fluid production. Notably, acupuncture combined with CHM exerts therapeutic effects gradually over the course of long-term treatment for the disease. In a clinical study (Low risk) that enrolled 94 pSS patients meeting the diagnostic criteria for the liver-kidney yin deficiency pattern, the participants were randomized into a treatment group and a control group. The treatment group undergo acupuncture at acupoints including Taiyang (EX-HN5), Jingming (BL1), and Chengqi (ST1), which was combined with an oral regimen of Erzhi Pill and Qiju Dihuang Pill. In contrast, the control group was administered oral Hydroxychloroquine sulfate tablets along with artificial tear eye drops. After 1 month of intervention, the treatment group exhibited more favorable outcomes than the control group across multiple parameters—estrogen, inflammatory markers, immunoglobulin, and both 5-minute tear and 15-minute saliva flow measurements—with a significantly higher total effective rate (85.4% vs. 69.6%, *P < 0.05*). The integrative protocol of Erzhi Pill, Qiju Dihuang Pill, and acupuncture is shown to promote tear and saliva secretion, which contributes to an improved quality of life for patients (150). 82 patients with SS (Some concerns) were divided into a Western medicine group which received oral Hydroxychloroquine Sulfate Tabletand and a TCM group which received oral Gui Bie enriching yin Powder combined with acupuncture at Taichong (LR 3), Taixi (KI 3), Quchi (LI 11), and other acupoints at random. After three months of treatment, the combination of Gui Bie Ziyin Powder and acupuncture was associated with notable improvements in ESSPRI, ESSDAI, IL-2, and sIL-2R levels. Additionally, the TCM group exhibited a total effective rate of 95.12%, a figure markedly exceeding the 73.17% recorded for the Western medicine group (*p < 0.05*). Consequently, the integrated therapy of Gui Bie Ziyin Powder and acupuncture demonstrates significant efficacy in alleviating the clinical symptomatology of SS (151). A total of 120 pSS patients (Some concerns) with yin deficiency and fluid depletion were randomly assigned to one of four treatment arms: 1) an integrated group comprising oral Yin-enhancing and fluid-producing decoction combined with acupuncture (at Lianquan-CV23, Xiaguan-ST7, Daying-ST5, etc.); 2) a Western medicine control receiving oral chloroquine; 3) a Chinese medicine control receiving the decoction alone; and 4) an acupuncture control receiving acupuncture alone. After 3 months of treatment, the acupuncture-medication treatment group showed significantly lower ESSPRI scores, level of IgG, Erythrocyte Sedimentation Rates(ESR), and scores for xerostomia, xerophthalmia, dry throat, dry cough, hot palms and soles, and dry skin compared to the other three groups. Meanwhile, the saliva flow rate and tear fiow in the acupuncture-medication treatment group were significantly higher than in the other three groups. The acupuncture-medication combination yielded a significantly higher total effective rate of 93.33%, outperforming the Western medicine (60.00%), Chinese herbal medicine (76.67%), and acupuncture-only (70.00%) control groups (*P<0.05*) (152). A total of 76 patients with SS were randomized into two arms in a randomized controlled trial (high risk). The 36 participants in the control arm received hydroxychloroquine sulfate monotherapy, whereas the 40 subjects in the treatment arm were given electroacupuncture plus modified Mume Pills in addition to the identical oral hydroxychloroquine sulfate regimen. Patients in the treatment group obtained notably better relief of TCM syndromes compared with controls, and this between-group disparity reached statistical significance (*P < 0.05*) following a 12-week intervention period. The Schirmer test results, salivary flow rate, CRP, IgG and ESR of patients in the treatment group were significantly improved compared with baseline (*P < 0.05*); only the Schirmer test results, salivary flow rate and CRP were improved in the control group (*P < 0.05*). Statistically significant intergroup differences *(P < 0.05*) were detected across all measured indicators, with superior outcomes observed in the treatment arm (153). Another randomized controlled (high risk) trial enrolled 60 SS patients, who were randomly assigned to a control group and an observation group with 30 cases each. Hydroxychloroquine sulfate tablets served as the sole intervention for subjects in the control group. By contrast, patients in the observation group underwent acupuncture therapy alongside modified Shashen Maidong Decoction. Both groups presented declines in IgG and ESR after 3 months of therapy relative to baseline (*P < 0.05*); further reductions of these biomarkers were identified in the observation group (*P < 0.05*). The TCM syndrome scores of both groups declined after treatment (*P < 0.05*). Compared with the control group, patients in the observation group achieved lower dry mouth and dry eye symptom scores together with a higher overall clinical efficacy rate. All discrepancies between the two groups reached statistical significance (*P < 0.05*) (154). In summary, small-scale clinical studies have demonstrated that acupuncture combined with traditional Chinese medicine exerts superior effects in nourishing yin, clearing heat, moistening dryness and promoting fluid production, effectively alleviating patients’ symptoms such as xerostomia and xerophthalmia, and presents advantages in the treatment of SS.

##### 5.1.3.2 Damp retention, heat stagnation, and collaterals obstructed by phlegm retention

Sjögren’s syndrome has an insidious onset and is characterized by stubbornness, often with relapses, leading to chronic disease entering the collaterals. A common therapeutic strategy is to activate blood and resolve stasis, while simultaneously tonifying qi and producing fluids. 60 patients with primary Sjögren’s syndrome free of visceral organ involvement were randomly assigned to two arms in a randomized controlled trial (Some concerns). Thirty participants received acupuncture combined with herbal medication, while the other 30 were placed in the Western medicine control arm. Patients in the combined therapy group received acupuncture (main acupoints: Hegu, Pishu, Shenshu, Zusanli, Sanyinjiao, Taixi; local auxiliary acupoints selected according to symptoms: Jingming, Cuanzu, Yuyao, Sizhukong, Yangbai and Sibai for severe dry eyes; Lianquan, Jinjin, Yuye and Jiache for severe dry mouth; Yingxiang for dry nose) combined with oral Jiedu Tongluo Shengjin Decoction. The control group was treated with hydroxychloroquine tablets. Superior therapeutic effects were seen in the treatment group following a 3-month treatment course, with a total effective rate of 83.33% compared to 60.00% recorded in the control group (*P < 0.05*). Significantly greater improvements in ESSPRI score, TCM syndrome score, salivary flow rate and ESR were observed among patients in the treatment arm relative to the control arm (*P < 0.05*). All assessed markers including ESSDAI, Schirmer’s test, immunoglobulins, complements and CRP showed statistically remarkable enhancements after therapy in patients from both arms (*P < 0.05*). Comparable therapeutic effects were achieved between the two arms, and no statistically meaningful intergroup disparities were observed regarding the alleviation of laboratory indices such as CRP, serum IgG and complements (*P > 0.05*) (155). Another randomized controlled trial (high risk) randomized 60 patients into two groups of 30 each. The control group received Zaobi Prescription, while the treatment group was given filiform needle acupuncture on the basis of the same herbal formula (main acupoints: Quze, Xuehai, Taichong, Sanyinjiao, Taixi; auxiliary acupoints: Shaoze, Lianquan, external Jinjin, external Yuye, Sibai). After 15 days of treatment, salivary flow rate and tear secretion volume were significantly improved in both groups after intervention. The total effective rate was 76.67% in the control group and 90% in the treatment group, indicating better outcomes in the treatment group (*P<0.05*) (156). In a clinical observational study (high risk), 60 pSS patients were divided into a treatment group and a control group at random. The control group received oral administration of the qi-tonifying and fluid-producing powder, while the treatment group undergoed acupuncture at points such as Lianquan (CV 23), Jingming (BL 1), and Yingxiang (LI 20) in addition to the other group’s treatment. A significantly greater reduction in the ESSPRI score, assessed by the EULAR Sjögren’s Syndrome Patient Reported Index, was observed in the treatment group compared to the control group after four weeks of treatment, along with superior relief from dryness and fatigue (*P < 0.05*). However, there were no significant abnormalities in immune indicators, saliva flow rate, or tear flow rate before and after treatment in the two groups (157). A RCTs (Some concerns) allocated 106 pSS patients into two parallel cohorts. The control cohort was administered Lu’s Runzao Decoction orally. In contrast, the therapeutic regimen for the intervention cohort was augmented with acupuncture at a predefined set of acupoints, including Zhongwan (CV 12), Sanyinjiao (SP 6), and Zusanli (ST 36), in addition to the identical herbal medication. Following the 3 months intervention, both therapeutic regimens demonstrated a comparable improvement in SS-related xerophthalmia and Schirmer’s test results, with no statistically significant intergroup difference observed in the latter. However, the treatment group exhibited superior efficacy in alleviating eye dryness, as evidenced by a significantly higher total effective rate on the VAS (67.92%) compared to the control group (54.72%; *P < 0.05*). These results confirm that acupuncture therapy has a certain short-term efficacy in alleviating the xerophthalmia of patients with pSS (158).

##### 5.1.3.3 Maintaining moisture, the stagnation of thermal activity, and the blockage of collateral pathways due to phlegm retention

A deficiency in yin-fluid constitutes the core pathological basis for the development of this condition. As yin deficiency gradually progresses, it is often combined with dampness, heat, phlegm, and stasis, and is commonly treated by clearing heat and dispelling dampness, and activating blood and resolving stasis. Chronic disease should not be rushed; a gentle treatment approach is required. For example, 141 female SS patients (Some concerns) were randomly divided into a Western medicine Control group (oral Hydroxychloroquine Sulfate Tablets), a Chinese medicine Control group (oral Linggui Zhugan Decoction), an acupuncture Control group (acupuncture at CV 23, ST 7, ST 5, and other points), and a combined acupuncture and herbal medicine treatment group (oral Linggui Zhugan Decoction combined with acupuncture). A 3 months clinical evaluation demonstrated that the integrated application of Linggui Zhugan Decoction and acupuncture yielded optimal therapeutic outcomes in the management of SS. This combined regimen was observed to effectively enhance the secretory capacity of both salivary and lacrimal glands, leading to a marked alleviation of characteristic clinical manifestations, including xerostomia, xerophthalmia, dry throat, and desiccated skin. Furthermore, the treatment was associated with statistically significant reductions in the ESSDAI score, as well as in serum levels of ESR and IgG, indicating a successful modulation of the underlying aberrant immune state (159). The integrated therapy group yielded a significantly higher total effective rate of 94.29% (*P < 0.05*), outperforming the control groups receiving acupuncture alone (60.00%), Chinese herbal medicine alone (65.71%), and Western medicine alone (61.11%).A randomized allocation (Some concerns) was performed to assign 86 pSS patients into two comparative cohorts: a control cohort receiving acupuncture monotherapy and an intervention cohort administered with an integrated regimen comprising oral Huayu Jiedu Decoction combined with adjunctive acupuncture therapy. The combination of Huayu Jiedu Decoction and acupuncture, as opposed to acupuncture therapy alone, can significantly improve symptoms such as xerophthalmia, xerostomia, and fatigue in pSS patients, reduce inflammatory reactions, and enhance immune function (160). This conclusion is supported by the study’s findings after 4 weeks, where the effective rate in the treatment group reached 95.35%, exceeding the 74.42% rate in the control group.

#### 5.1.4 Acupuncture combined with integrated traditional Chinese and western medicine treatment

Acupuncture combined with oral administration of both TCM and Western medicine is typically indicated for patients in the acute phase of the disease, characterized by markedly elevated inflammatory markers and severe clinical symptoms such as xerostomia, xerophthalmia, and pain. As the root cause of this disease is yin deficiency, prolonged unresolved illness or delayed treatment may lead to yin damage affecting yang, resulting in deficiency of both yin and yang. Accordingly, treatment for this dual deficiency should prioritize enriching yin to generate fluid and warming yang to transform qi. If the disease does not heal for a long time or treatment is delayed, yin damage may affect yang, leading to deficiency of both yin and yang. The treatment should focus on enriching yin and producing fluid, warming yang and transforming qi. A representative clinical trial (Some concerns) involve 113 SS patients, who were allocated to either a standard care group or a combination therapy group. The standard care group received monotherapy with oral Hydroxychloroquine Sulfate Tablets. In comparison, the combination therapy group was administered a modified Gualou Qumai Decoction regimen supplemented with acupuncture stimulation at bilateral BL 23, KI 3, and LR 3 acupoints as adjunctive treatment. Upon completion of the 2 months therapeutic course, a statistically significant disparity in clinical efficacy was observed between the two cohorts (*P<0.05*). The observation group demonstrated a markedly superior effective rate of 96.49%, compared to 85.71% in the control group. Furthermore, comprehensive assessment revealed that patients receiving combination therapy showed significant improvements across multiple clinical parameters, including:① TCM syndrome manifestations (xerostomia, xerophthalmia, ocular dryness, cutaneous desiccation, etc.);② standardized disease activity metrics; ③ objective secretory function (salivary flow and Schirmer’s test); and ④ serological markers of inflammation and immune response (immunoglobulin profiles, ESR, and CRP), all indicating a more favorable therapeutic profile relative to the control group. Therefore, the clinical effect of Hydroxychloroquine Sulfate Tablets combined with modified Gualou Qumai Decoction and acupoint acupuncture in the treatment of SS is significant and has high safety (161). A randomized clinical (Some concerns) investigation enrolled 96 primary pSS patients who were allocated to either a conventional therapy group or an integrated intervention group. Participants in the conventional therapy group received monotherapy with Total Glucosides of Radix Paeoniae Alba Capsules, whereas those in the integrated intervention group were administered Suangan Granules as an adjunct to the conventional treatment, supplemented with acupuncture stimulation at designated acupoints including ST 4 (Dicang), ST 6 (Jiache), and KI 3 (Taixi). A 2 months therapeutic intervention revealed statistically significant intergroup differences in both laboratory parameters and clinical outcomes. The combination therapy group exhibited markedly greater reductions in inflammatory and immunologic markers—including ESR, CRP, and IgG—relative to the control group. Clinical efficacy was further demonstrated by a substantially higher overall treatment response rate in the combination group (93.8%) compared to the control group (58.3%). These findings collectively indicate that the integrated protocol of Total Glucosides of Paeonia Capsules, Suangan Granules, and acupuncture produces a clinically meaningful synergistic effect in managing pSS (162).

#### 5.1.5 Acupuncture combined with other therapies

In addition to the methods mentioned above, acupuncture combined with other therapies also has good effects in treating Sjögren’s syndrome and therapeutic methods include bloodletting, pricking and cupping, and kinesiotherapy. One randomized controlled trial (Some concerns) randomly divided 40 patients with Sjögren’s syndrome into two groups of 20 each. The treatment group received collateral pricking blood therapy combined with acupuncture, while the control group was treated with conventional acupuncture. Based on the acupoints used in the control group (Baihui, Yintang, Yingxiang, Lianquan, Hegu, Zusanli, Taixi, Sanyinjiao, Xuehai), the treatment group additionally received pricking bloodletting at Jinjin and Yuye. After 50 days of treatment, the total clinical effective rate was 85.0% in the treatment group and 50.0% in the control group, with a statistically significant difference between groups (*P < 0.05*) (163). Another randomized controlled trial (Some concerns) allocated 72 patients with xerophthalmia to two groups. Patients within the treatment group underwent acupuncture therapy; the selected acupoints consisted of Baihui, Jingming, Cuanzhu, Taiyang, Sibai, Fengchi and other auxiliary points. And combined with thunder-fire moxibustion: circling moxibustion around both eyes first, followed by sparrow-pecking moxibustion at Jingming, Cuanzhu, Taiyang, Sibai, Fengchi and Hegu for 2–3 minutes per eye, three times a week with 12 sessions as one course of treatment. The control group was administered artificial tears. After 8 weeks of intervention, ocular dryness, asthenopia, Schirmer test results and tear film break-up time were all improved in both groups compared with baseline (*P < 0.05*), and the treatment group achieved superior efficacy to the control group (*P < 0.05*) (164). Although two-thirds of SS patients experience symptoms of chronic fatigue, which severely impacts their quality of life (165), the clinical focus is often placed on addressing the issues of mucocutaneous dryness and painful joints in SS patients, while fatigue does not receive sufficient attention.

#### 5.1.6 Other acupuncture methods

Other acupuncture modalities include laser acupuncture and auricular press needles. Laser acupuncture may serve as an effective therapeutic approach for improving salivary flow rates in patients with SS, as suggested by a prospective, randomized, placebo-controlled study (Some concerns) (166). A total of 26 patients with grade III xerostomia were enrolled in this trial and randomly assigned to either a laser acupuncture treatment group (n=14) or a placebo laser acupuncture group (n=12). Laser acupuncture was administered via non-invasive stimulation at acupoints LI 2, ST 5, ST 6, ST 7, SI 19, and BL 13. The placebo group underwent an identical procedure, with the same device applied to the same acupoints for the same duration, with the exception that no radiation was emitted, although the audible signal remained active. Ultimately, saliva production increased significantly in the treatment group and was statistically greater than in the placebo group. However, the effect of laser acupuncture is time-sensitive, remaining consistently good at 3 months post-treatment but beginning to decline slightly by the 6th month. One study (167) (Some concerns) assigned 66 pSS patients presenting with yin fluid deficiency pattern to either a conventional treatment cohort or an augmented intervention cohort. All participants received a modified Qingzao Bujin Decoction regimen, with the augmented intervention cohort additionally undergoing auricular thumb-tack needle therapy targeting specific zones including the Eye, Mouth, and Liver acupoints. After 12 weeks, the effective rate for the primary efficacy indicator, TCM syndrome, in the experimental group was 87.10%, which was superior to the 73.33% in the Control group (*P < 0.05*). Statistical analyses revealed significant between-group differences in multiple patient-reported outcomes following the intervention period. The experimental cohort demonstrated statistically superior results (*P < 0.05*) across several validated assessment tools, including Traditional Chinese Medicine syndrome scores, ESSPRI measurements, SAS and FSS ratings, and PSQI evaluations. No statistically meaningful disparities (*P > 0.05*) between the two arms were observed in all objective physiological indices, which covered Schirmer’s test outcomes, resting salivary flow rates, IgG concentrations and SDS scores. The results suggest that auricular point thumb-tack needle therapy combined with modified Qingzao Bujin Decoction can not only effectively alleviate symptoms such as dryness of the mouth and eye in pSS but also improve patients’ sleep, depression, and fatigue.

Due to space limitations, only detailed data of partial studies are presented in the following text; detailed information regarding the rest of the studies is presented in **Table 3**.

**Table 3:** Characteristics of included studies not displayed in the text.

Author	Study Design	Participants	Intervention	Treatment Duration	Acupoints	Outcome Indicators	Safety Assessment
Li, J.Y. (183)	Prospective interventional study	34	Acupuncture	15 days	Chengqi (ST1), Sibai (ST2), Jingming (BL1), Taiyang (EX-HN5), Hegu (LI4)	a;b;c	N
Cao, R. (184)	Prospective interventional study	35	Acupuncture	2 months	Fengfu (GV16), Fengchi (GB20), Sanyinjiao (SP6), Neiting (ST44), Xingjian (LR2), Xuehai (SP10), Geshu (BL17), Jingming (BL1)	a;d;e;f	N
Qi, F.X. (185).	Prospective interventional study	30	Acupuncture	2 months	Lianquan (CV23), Zhongwan (CV12), Qihai (CV6), Bilateral Lieque (LU7), Bilateral Yuji (LU10), Bilateral Zhaohai (KI6)	h;a;g;n	N
Zhou, X.Y. (186)	Single-blind RCT	Treatment :60 ; Control : 60	Treatment: Acupuncture; Control: Sham acupuncture	2 months	Taiyang (EX-HN5), Cuanzhu (BL2), Sizhukong (TE23), Chengjiang (CV24), Jiache (ST6), Lianquan (CV23), Zhaohai (KI6), Waiguan (TE5)	a;o;i;j;k;g;p;q;d;e;l	Y
Lin, H. C. (187)	Single-blind RCT	GB group: 40 ; GBL group: 40 ; Control group: 20	GB group: Acupuncture at GB20 ; GBL group: Acupuncture at GB20+BL2; Control: No intervention	2 months	Fengchi (GB20), Cuanzhu (BL2)	k;r;i;b;s	Y
Qin, G.H. (188)	Prospective interventional study	20	Acupuncture	/	Dry eye special acupoint	t;k;s	N
Ma, B.D. (189)	Single-blind RCT	Treatment: 24 Control: 22	Treatment: Acupuncture; Control: Hydroxychloroquine	1 month	Middle Jiao area, Lower Jiao area, Liver reflex zone, Spleen reflex zone, Kidney reflex zone (ocular acupuncture zones)	a;d;t	N
Zhang, D. (190)	Single-blind RCT	Treatment: 38 Control: 38	Treatment: Acupuncture + Electroacupuncture; Control: Conventional acupuncture	1 month	Cuanzhu (BL2), Taiyang (EX-HN5), Sizhukong (TE23), Sibai (ST2), Fengchi (GB20), Baihui (GV20), Hegu (LI4), Zusanli (ST36), Guangming (GB37), Sanyinjiao (SP6), Taichong (LR3)	r;b;v;p	Y
Zhang,Y.C. (191)	RCT (no blinding)	Treatment: 30 ; Control: 31	Treatment: Acupuncture; Control: Artificial tears	20 days	Baihui (GV20), Jingming (BL1), Cuanzhu (BL2), Taiyang (EX-HN5), Sibai (ST2), Fengchi (GB20), Hegu (LI4), Zusanli (ST36), Sanyinjiao (SP6), Taixi (KI3), Yongquan (KI1)	b;k	N
Liu, J.W (192)	Single-blind RCT	Treatment: 28; Control: 28	Treatment: "Two Dragons Playing Pearl" + "Eye Warming" acupuncture manipulation; Control: Twisting reinforcing acupuncture manipulation	1 month	Cuanzhu (BL2), Sizhukong (TE23), Taiyang (EX-HN5), Fengchi (GB20), Feishu (BL13), Chize (LU5)	u;b;r	N
Liu, W (193)	Single-blind RCT	Treatment: 60 ; Control: 60	Treatment: Acupuncture; Control: Prednisone + Artificial tears	3 months	Quze (PC3), Taichong (LR3), Xuehai (SP10), Sanyinjiao (SP6), Taixi (KI3), Shaoze (SI1), Lianquan (CV23), External Jinjin, External Yuye, Jingming (BL1), Sibai (ST2), Jiache (ST6), Yifeng (SJ17)	a;k;g;e;f;b	Y
Du, G.S. (194)	RCT (no blinding)	Treatment: 40; Control: 40	Treatment: Acupuncture; Control: Hydroxychloroquine + Transfer factor oral liquid + Multivitamin + Pilocarpine + Lubricating eye gel	2 months	Jingming (BL1), Lianquan (CV23), Quze (PC3), Qihai (CV6), Xuehai (SP10), Sanyinjiao (SP6), Taixi (KI3), Taichong (LR3)	a;e;d;t	N
Bai, H. (195)	Single-blind RCT	Treatment: 30; Control: 27	Treatment: Electroacupuncture; Control: Hydroxychloroquine + Transfer factor oral liquid + Vitamin B + Pilocarpine	2 months	Shenshu (BL23), Taixi (KI3), Hegu (LI4), Sanyinjiao (SP6), Xuehai (SP10), Lianquan (CV23), Baihui (GV20)	a;x	N
Tong, J.Y. (179)	RCT (no blinding)	Treatment: 43; Control: 43	Treatment: Hydroxychloroquine + Bromhexine hydrochloride + Acupuncture; Control: Hydroxychloroquine + Bromhexine hydrochloride	2 months	Waiguan (TE5), Zhaohai (KI6), Sizhukong (TE23), Chengqi (ST1), Jiache (ST6), Taiyang (EX-HN5), Cuanzhu (BL2), Lianquan (CV23)	a;y;z;g;k	N
Pang, Y.J. (196)	RCT (no blinding)	Treatment: 30 ; Control: 30	Treatment: Acupuncture; Control: Artificial tears	1 month	Sibai (ST2), Cuanzhu (BL2), Chengqi (ST1), Yingxiang (LI20), Baihui (GV20), Shaoze (SI1), Houxi (SI3)	u;b;m	N
Yang, G.Y. (197)	Prospective interventional study	34	Acupuncture combined with salivary gland duct injection of Dexamethasone	1 month	Fengfu (GV16)	a;g;k	N
Deng, S.X. (198)	Single-blind RCT	Treatment: 33; Control: 33	Treatment: Artificial tears + Glucocorticoid + Immunosuppressant + Acupuncture; Control: Artificial tears + Glucocorticoid + Immunosuppressant	14 weeks	Yanglao (SI6)	i;y;v;b;k;d;l;e;I	Y
Zheng, X.L. (199)	Single-blind RCT	Treatment: 61; Control: 61	Treatment: Hydroxychloroquine + Total glucosides of paeony + Methotrexate + Acupuncture + Yiguanjian Decoction; Control: Hydroxychloroquine + Total glucosides of paeony + Methotrexate + Acupuncture	3 months	Taixi (KI3), Taichong (LR3), Zusanli (ST36), Sanyinjiao (SP6), Hegu (LI4), Chengjiang (CV24), Lianquan (CV23), Jiache (ST6), Xiaguan (ST7), Yifeng (TE17), Jingming (BL1), Sibai (ST2), Cuanzhu (BL2), Yuyao (EX-HN4), Sizhukong (TE23)	j;g;l;d	N
Kou J.Y. (200)	Prospective interventional study	25	Acupuncture combined with Runwen Fuge Decoction	1 month	Qianzheng (EX-HN16), Jinjin, Yuye, Jingming (BL1), Pishu (BL20), Shenshu (BL23), Mingmen (GV4), Sanyinjiao (SP6)	o;II	N
Deng, J.H. (180).	Single-blind RCT	Treatment: 55; Control: 54	Treatment: Lu's Runzao Decoction + Acupuncture; Control: Lu's Runzao Decoction	3 months	Bilateral Gongsun (SP4), Bilateral Sanyinjiao (SP6), Bilateral Zusanli (ST36), Bilateral Hegu (LI4), Bilateral Neiguan (PC6), Zhongwan (CV12), Bilateral Jiache (ST6)	h;g	N
Zhang, J.H. (201)	Single-blind RCT	Acupuncture group:25; TCM group:25; Acupuncture+TCM group:25; Western medicine group:25	Acupuncture: Acupuncture; TCM: Banban Yangyin Granules; Acupuncture+TCM: Acupuncture + Banban Yangyin Granules; Western medicine: Hydroxychloroquine sulfate	3 months	Cuanzhu (BL2), Sizhukong (TE23), Jiache (ST6), Chengjiang (CV24), Lianquan (CV23), Ganshu (BL18), Shenshu (BL23), Zusanli (ST36), Sanyinjiao (SP6), Taixi (KI3)	a;i;j;g;k;e;d;l	Y
Fan, J.Y. (202)	Single-blind RCT	Acupuncture+TCM:38; TCM:38; Western medicine:38	Acupuncture+TCM: Electroacupuncture + Modified Biejia San; TCM: Modified Biejia San; Western medicine: Hydroxychloroquine sulfate tablets	3 months	Jiache (ST6), Quanliao (SI18), Xiaguan (ST7), Yifeng (TE17)	y;j;i;h;g;v;k;d;l;e;II	Y
Yang, X.Y. (203)	Single-blind RCT	Treatment: 42; Control: 42	Treatment: Basic TCM formula + Acupuncture; Control: Methotrexate	3 months	Sibai (ST2), Yuyao (EX-HN4), Hegu (LI4), Jingming (BL1), Lianquan (CV23), External Jinjin, External Yuye, Jiache (ST6), Zusanli (ST36)	d;l;e;k;g	N
Li, Z. (204)	Prospective interventional study	20	Acupuncture combined with Chinese herbal decoction	3 months	Qihai (CV6), Guanyuan (CV4), Bilateral Tianshu (ST25), Bilateral Guilai (ST29), Bilateral Ququan (LR8), Bilateral Sanyinjiao (SP6), Bilateral Taixi (KI3), Bilateral Chize (LU5)	v;h;d;y	Y
Cong, Y. (205)	RCT (no blinding)	Treatment: 21; Control: 21	Treatment: Yiqi Shengjin Huoxue Decoction + Acupuncture; Control: Yiqi Shengjin Huoxue Decoction	/	Taichong (LR3), Quze (PC3), Sanyinjiao (SP6), Xuehai (SP10), Taixi (KI3), Lianquan (CV23), Cuanzhu (BL2), Sizhukong (TE23)	a;c;g	N
Gu, J.H. (206)	Single-blind RCT	Acupuncture+TCM:30; TCM:30; Western medicine:30	Acupuncture+TCM: Shugan Shengjin Decoction + Acupuncture; TCM: Shugan Shengjin Decoction; Western medicine: Hydroxychloroquine	3 months	Taichong (LR3), Hegu (LI4), Sanyinjiao (SP6), Zusanli (ST36), Taixi (KI3), Xuehai (SP10)	l;f;e;d;k;t;y;a;II	Y
Gu, Q. (207)	Prospective interventional study	48	Filiform needle acupuncture + Oral Chinese herbal medicine	1 month	Hegu (LI4), Lianquan (CV23), Shenshu (BL23), Zusanli (ST36), Sanyinjiao (SP6), Taixi (KI3)	a;e;d;IV;V	N
Hu, L.P. (208)	RCT (no blinding)	Treatment: 30; Control: 30	Treatment: Zaobi Standard Formula + Acupuncture; Control: Zaobi Standard Formula	15 days	Taichong (LR3), Quze (PC3), Sanyinjiao (SP6), Xuehai (SP10), Taixi (KI3), External Yuye, Shaoze (SI1), Sibai (ST2), Lianquan (CV23), External Jinjin	h;v;k;g;a	N
Zhang, B.G. (209)	RCT (no blinding)	Treatment: 43; Control: 42	Treatment: Hydroxychloroquine sulfate + Ocular acupuncture + Chaihu Guizhi Ganjiang Decoction; Control: Hydroxychloroquine sulfate	1 month	Ocular acupuncture zones: Upper Jiao zone, Liver-Gallbladder zone, Middle Jiao zone, Spleen-Stomach zone, Lung zone, Kidney zone	a;y;i;j;o	N
Yang, D. (181)	Single-blind RCT	Treatment: 45; Control: 45	Treatment: Cyclopentthiolate + 0.2% Carbomer eye gel + Yinyin Decoction combined with Yiguanjian Decoction + Acupuncture; Control: Cyclopentthiolate + 0.2% Carbomer eye gel	3 months	Dry eye special acupoint	v;c;m;b	N
Li, X. (182)	Single-blind RCT	Treatment: 30; Control: 30	Treatment: Hydroxychloroquine Sulfate Tablets + Total Glucosides of Paeony Capsules + Intradermal Needle + Chinese Herbal Fumigation; Control: Hydroxychloroquine Sulfate Tablets + Total Glucosides of Paeony Capsules	1 month	Cuanzhu (BL2), Jingming (BL1), Taiyang (EX-HN5), Zusanli (ST36), Sanyinjiao (SP6), Sizhukong (TE23), Fengchi (GB20), Sibai (ST2), Ganshu (BL18), Shenshu (BL23)	y;e;d;f;r;ub	Y
Ou, Y. J. (210)	RCT (no blinding)	Treatment: 20; Control: 6	Treatment: Filiform acupuncture + Herbal decoction + Total glucosides of paeony capsules + Hydroxychloroquine; Control: Total glucosides of paeony capsules + Hydroxychloroquine	1 month	Bilateral Hegu (LI4), Bilateral Jiache (ST6), Bilateral Sanyinjiao (SP6), Bilateral Taixi (KI3)	a;o;d;l	N
Wang, H.Q. (211)	RCT (no blinding)	Treatment: 30; Control: 30	Treatment: Hydroxychloroquine sulfate + Ziyin Runzao Decoction + Acupuncture; Control: Hydroxychloroquine sulfate + Total glucosides of paeony capsules	2 months	Jinjin, Yuye	a;o;d;e;l;k;g	Y
Cheng, X.F. (212)	Single-blind RCT	Treatment: 20; Control: 20	Treatment: Acupuncture + Herbal decoction + Hydroxychloroquine sulfate; Control: Herbal decoction + Hydroxychloroquine sulfate	1 month	Bilateral Sanyinjiao (SP6), Bilateral Xuehai (SP10), Bilateral Hegu (LI4), Bilateral Taichong (LR3), Bilateral Sizhukong (TE23), Bilateral Yangbai (GB14), Bilateral Sibai (ST2), Lianquan (CV23), Bilateral Zusanli (ST36), Bilateral Yinlingquan (SP9), Bilateral Taixi (KI3)	l;g;k;o;i;j;y;a	Y
Ma, L. (213)	Prospective interventional study	35	Acupuncture combined with acupoint injection (Qingkailing injection or Vitamin B12 / Vitamin B1 injection)	2 months	Liangqiu (ST34), Zusanli (ST36), Xuehai (SP10), Dadu (SP2), Yuji (LU10), Shaoshang (LU11), Wenliu (LI7), Quchi (LI11), Taichong (LR3), Quze (PC3), Guangming (GB37), Zulinqi (GB41), Rangu (KI2), Sanyinjiao (SP6), Feiyang (BL58), Jinggu (BL64), Fengfu (GV16), Chengjiang (CV24), Lianquan (CV23)	o	N
Si, X.W. (214)	RCT (no blinding)	Treatment: 38; Control: 20	Treatment: Chinese herbal ear point therapy; Control: Vitamin A, Vitamin E + Prednisone + Artificial tears + Acyclovir eye drops	4 months	Ear points: Liver, Spleen, Lung, Kidney, Heart, Eye, Endocrine	a;m;k	N

NOCT: a:clinicalefficacy; b:tear film break-uptime(BUT); c:tearsecretionvolume; d:erythrocytesedimentationrate(ESR); e:immunoglobulin(Ig); f:rheumatoidfactor (RF); g:salivary flow rate; h:dry mouthVASscore; i:EULAR Sjögren’s Syndrome Patient Reported Index (ESSPRI); j:EULAR Sjögren’s Syndrome Disease Activity Index (ESSDAI); k:Schirmertest; l:C-reactive protein (CRP) C; m:corneal fluorescein staining; n:dry mouth symptom score; o:VAS score for dryness, limb pain and fatigue; p:Quality of life index based on the 36-item Medical Outcomes Study Short- Form Health Survey (SF-36) in EULAR Sjögren’s syndrome cohorts; q:Hospital Anxiety and Depression Scale (HADS); r:Ocular Surface Disease Index (OSDI); s:artificial tear usage; t:main symptom integral; u:STT-1; v:ocular dryness VAS score; w:quality of life evaluation x hormone levels: progesterone, estradiol, testosterone, follicle-stimulating hormone (FSH), luteinizing hormone (LH), prolactin; y:changes in TCM syndrome integral; z:emotional changes. I: Onset time of acupuncture effect and duration of therapeutic efficacy; II: sugar cube test score; III: salivary gland ultrasound score; IV: fibrinogen (FIB); V: gammaglobulin γ.

RCT, Randomized Controlled Trial; Y, Yes; N, no.

### 5.2 Safety considerations of acupuncture for Sjögren’s syndrome

Acupuncture is widely applied in clinical practice at present (168, 169) and is increasingly integrated into routine treatment regimens. Nevertheless, some scholars have long questioned its safety and criticized its insufficient therapeutic efficacy and inadequate scientific evidence (170). As early as 1995 (171), researchers summarized all case reports published between 1966 and 1993 and identified a total of 395 adverse events associated with acupuncture. Most complications were mild, such as ecchymosis or vasovagal syncope, while 216 events were severe, including several cases of pneumothorax and spinal cord injury. The authors of that report noted that most adverse incidents documented in the case series could have been completely prevented if the procedures had been performed by competent practitioners.

A prospective survey conducted in the United Kingdom (172) concluded that the overall incidence of adverse events among 66,229 patients was 10.2%. The most frequent reactions included fatigue (3%), bleeding or bruising (3%), transient exacerbation of original symptoms (2%), and local pain at needle insertion sites (1%), with no severe adverse events recorded. This paper sorts out and summarizes the adverse events of clinical studies included in Section 3 (see **Table 4** for details). According to the data in the table, the main adverse reactions of acupuncture for Sjögren’s syndrome are local ecchymosis and pain at acupoints. No severe adverse events such as syncope, infection or organ injury were observed with a low overall incidence rate, indicating that acupuncture is relatively safe for treating Sjögren’s syndrome in clinical practice. Nevertheless, it should be noted that only a small proportion of the included studies documented adverse reactions, and some reports had vague descriptions, which hinders an objective and comprehensive evaluation of the actual incidence of acupuncture-related adverse events. Future relevant clinical trials should standardize the recording and reporting of adverse events, fully disclose all mild and severe discomforts, and provide sufficient and reliable data to support the safe clinical application of acupuncture for Sjögren’s syndrome.

**Table 4:** The record of adverse events.

Trial	Mentioned	Treatment group adverse reactions	Control group adverse reactions
List, T. (10)	YES	Burning sensations in the mouth and tongue were reported, without specific grouping information provided	/
Zhou, X. (84)	YES	1 case of upper limb fracture, 1 case of uterine fibroids	1 case of dental neuralgia, 1 case of lower limb fracture
Liu, W. (87)	YES	No adverse reactions occurred	28 cases of central obesity, 6 cases of hypertension, 4 cases of elevated blood glucose, 4 cases of insomnia
Gomes-Silva, J.M. (136)	YES	Small bruises and mild transient fatigue were reported, without specific grouping information provided	/
Qi, W.W. (138)	NO	/	/
Wang, S. (139)	YES	1 case of insomnia	2 cases of central obesity, 3 cases of elevated blood glucose, 2 cases of insomnia
Xu, D.K. (145)	YES	No adverse reactions occurred	15 cases of loose stools
Lan, Y. (143)	YES	1 patient experienced severe facial distension and numbness, lasting for about 2 hours	No obvious adverse reactions observed
Wang, C. (144)	YES	1 case of subcutaneous ecchymosis after acupuncture	1 case of mild gastrointestinal discomfort
Lai, L.S. (149)	NO	/	/
Ren, J.B. (150)	NO	/	/
Gao, Z.Q. (151)	YES	1 case of mild liver function abnormality, 1 case of blurred vision	2 cases of loose stools, 3 cases of mild liver function abnormality, 2 cases of blurred vision, 1 case of nausea and vomiting, 2 cases of dizziness
Xu, D.K. (152)	YES	No adverse events occurred	1 case of subcutaneous ecchymosis
Chen, A.P. (157)	YES	1 patient could not tolerate acupuncture pain, 2 cases of bleeding at acupuncture sites	1 case of respiratory tract infection
Ge, L. (158)	NO	/	/
Hou, X.S. (159)	YES	2 cases of skin pruritus, 1 case of nausea and vomiting, 1 case of suppressed tendon reflex, 1 case of skin ecchymosis	Western medicine control group: 1 case of skin pruritus, 1 case of nausea and vomiting, 1 case of dizziness; Traditional Chinese medicine control group: 1 case of skin pruritus, 1 case of nausea and vomiting; Acupuncture control group: 1 case of nausea and vomiting, 1 case of suppressed tendon reflex, 1 case of skin ecchymosis
Zhai, J.L. (160)	NO	/	/
Xing, G.E. (161)	YES	No adverse reactions occurred	No adverse reactions occurred
Ge, H.Q. (162)	NO	/	/
Luo, Y.W. (167)	YES	1 case of auricular skin erythema, 1 case of mild ear desquamation, 1 case of mild ear pain, 1 case of mild abdominal distension and loose stool	2 cases of mild nausea and abdominal distension
Cafaro, A. (166)	YES	No adverse reactions occurred	No adverse reactions occurred
Li, J.Y. (183)	NO	/	/
Ren, B. (140)	YES	No adverse reactions occurred	No adverse reactions occurred
Zhang, X.L. (141)	YES	No adverse reactions occurred	14 cases of central obesity, 3 cases of hypertension, 2 cases of elevated blood glucose, 2 cases of insomnia
Cui, X.C. (142)	NO	/	/
Feng, G.Y. (153)	YES	2 cases of gastric discomfort, 1 case of mild elevated liver enzymes, 1 case of drug withdrawal, 1 case of subcutaneous bleeding	1 case of gastric discomfort, 1 case of mild elevated liver enzymes
Li, B. (155)	YES	1 patient developed scalp rash accompanied by pruritus	1 patient had slight blurred vision when initially taking hydroxychloroquine tablets
Chen, F.Y. (156)	NO	/	/
Tan, Y. (164)	NO	/	/
Liu, S.H. (163)	NO	/	/
Wang, X.C. (154)	NO	/	/
Cao, R. (184)	NO	/	/
Qi, F.X. (185)	NO	/	/
Zhou, X.Y. (186)	YES	2 patients presented with ecchymosis and pain at acupuncture sites, 1 patient had local pruritus at the patch application area	2 patients reported pain
Lin, H. C. (187)	YES	Pain and ecchymosis were mentioned, without detailed information specified	/
Qin, G.H. (188)	NO	/	/
Ma, B.D. (189)	NO	/	/
Zhang, D. (190)	YES	1 case of ecchymosis at Taiyang acupoint, 1 case of ecchymosis at Fengchi acupoint	2 cases of ecchymosis at Cuanzhu acupoint
Zhang, Y.C. (191)	NO	/	/
Liu, J.W. (192)	NO	/	/
Liu, W. (193)	YES	No adverse reactions occurred	28 cases of central obesity, 6 cases of hypertension, 4 cases of elevated blood glucose, 4 cases of insomnia
Du, G.S. (194)	NO	/	/
Bai, H. (195)	NO	/	/
Tong, J.Y. (179)	NO	/	/
Pang, Y.J. (196)	NO	/	/
Yang, G.Y. (197)	NO	/	/
Deng, S.X. (198)	YES	1 case of subcutaneous ecchymosis	No adverse reactions occurred
Zheng, X.L. (199)	NO	/	/
Kou, J.Y. (200)	NO	/	/
Deng, J.H. (180)	NO	/	/
Zhang, J.H. (201)	YES	No adverse reactions occurred	No adverse reactions occurred
Fan, J.Y. (202)	YES	1 patient reported pain, 1 case of ecchymosis at acupuncture site	Traditional Chinese medicine group: 1 patient complained of loose stool after medication; Western medicine group: 1 patient complained of dizziness
Yang, X.Y. (203)	NO	/	/
Li, Z. (204)	YES	No adverse reactions occurred	No adverse reactions occurred
Cong, Y. (205)	NO	/	/
Gu, J.H. (206)	YES	No adverse reactions occurred	No adverse reactions occurred
Gu, Q. (207)	NO	/	/
Hu, L.P. (208)	NO	/	/
Zhang, B.G. (209)	NO	/	/
Yang, D. (181)	NO	/	/
Ou, Y.J. (210)	NO	/	/
Wang, H.Q. (211)	YES	1 case of soft loose stool, 1 patient presented with abdominal distension and discomfort after medication, without grouping information clarified	/
Cheng, X.F. (212)	YES	2 cases of upper respiratory tract infection, 1 case of elevated liver function indicators	3 cases of leukopenia, 2 cases of elevated liver function indicators
Ma, L. (213)	NO	/	/
Si, X.W. (214)	NO	/	/

From a safety perspective, the popularization of single-use sterile acupuncture needles has markedly reduced the risk of infection linked to acupuncture treatment. However, it is worth noting that a proportion of SS patients receive immunosuppressive agents (173), and some patients develop primary Sjögren’s syndrome-related immune thrombocytopenia (ITP) (174), both of which raise the risks of bleeding and infection (175). Accordingly, clinicians must conduct a thorough physical assessment of patients prior to acupuncture manipulation to avoid severe adverse events.

In the studies included in this research on acupuncture treatment for SS patients, no studies mentioned the occurrence of serious adverse events. The described adverse events were mostly diarrhea, bruising at the acupuncture site, etc. However, some studies did not record adverse events, which imposed certain limitations on the assessment of the safety of acupuncture treatment for SS and could lead to biases in the evaluation of efficacy and safety results.

## 6 Discussion

Sjögren’s syndrome (SS), an immune-mediated disorder, is primarily characterized by xerostomia and xerophthalmia, which are its hallmark symptoms. Its etiology remains elusive, being potentially associated with factors including immune dysregulation, hormonal imbalance, viral infections, and emotional status. As a core non-pharmacological intervention in traditional Chinese medicine (TCM), acupuncture boasts promising prospects for the treatment of rheumatic immune diseases such as rheumatoid arthritis (176), acute gouty arthritis (177) and knee osteoarthritis (178). Meanwhile, acupuncture is increasingly adopted as an adjuvant therapy for Sjögren’s syndrome (SS), and substantial clinical evidence has validated its therapeutic efficacy. Acupuncture may exert its therapeutic effects through the following mechanisms: ① regulating pro-inflammatory factors through the cholinergic anti-inflammatory pathway; ② regulating immunoglobulins; ③regulating the imbalance between Th17 and Treg; ④regulating the innate immune response; ⑤regulating the intestinal microbiota. According to the data in **Table 1**, the research and discussion in this review on the mechanism of acupuncture in treating Sjögren’s syndrome are mostly based on speculative hypotheses and comprehensive retrospective analyses. Direct evidence in SS patients is still limited, and there is a lack of targeted and systematic mechanism studies - this may be related to the unclear etiology of SS itself, but it is also a deficiency of this study and one of the directions that deserve further research in the future.

In the management of Sjögren’s syndrome, acupuncture is frequently incorporated into integrative approaches. Common combinatorial regimens include its concurrent application with traditional Chinese herbal medicine, its adjunctive use alongside Western pharmaceutical agents, and its role within comprehensive frameworks that synergize both traditional and modern medical principles. Regardless of the treatment method, the key to achieving good results lies in the ability to quickly alleviate the condition in the present. Therefore, the crucial factor for achieving good results is the clinician’s ability to accurately assess the patient’s condition and the stage of the disease, thereby selecting the appropriate treatment method. This ensures that the medication’s potency is neither excessive (avoiding hormone abuse) nor insufficient (preventing irregular use of Western medicine), and, more importantly, that the treatment is correctly targeted. Only in this way can the progression of Sjögren syndrome be better controlled. Clinicians must not rigidly adhere to others’ successful experiences by applying them without understanding flexibility. In clinical scenarios involving acute inflammatory exacerbations, therapeutic strategies should not rely exclusively on traditional Chinese medicine modalities, even when certain clinical evidence suggests superior efficacy of acupuncture monotherapy or acupuncture-herbal combinations over conventional Western treatments. Instead, appropriate Western medical interventions should be selected to control the imbalanced immune response. In clinical practice, doctors need to grasp the essence of the disease: treating symptoms urgently when necessary and addressing the root cause when possible, in order to effectively resolve the problem.

By reviewing current “ Practical Clinical Application of Acupuncture in Managing Sjögren’s Syndrome.,” the following characteristics are summarized:①Most studies are domestic research without ethics approval and clinical trial registration. They failed to conduct sample size calculation, adopt blinding methods, or clearly describe allocation concealment, and some studies carry a high risk of bias; ② Most current trials comparing acupuncture groups with blank control groups are prospective studies, while randomized controlled trials remain insufficient. The absence of rigorous randomization and controlled designs weakens the strength of their evidence. ③ Some studies have treatment courses of 4 weeks, 8 weeks, or 1 month, which are relatively short; ④Most studies lack a follow-up process, making it impossible to observe and evaluate long-term efficacy; ⑤ The vast majority of studies lack standardized and appropriate primary efficacy indicators, and when primary efficacy indicators mention the clinical efficacy of TCM syndromes, they are devoid of internationally recognized professional evaluation and valid evidence.⑥ Some studies lack descriptions of adverse events, and the results of safety evaluations are biased. In summary, current clinical studies lack standardization, are of relatively low quality, and some clinical trial designs do not strictly adhere to the principles of randomization, control, replication, and blinding. In some trials with true acupuncture versus sham acupuncture control, the design of experimental and control groups also lacks a certain degree of rigor.

A randomized controlled trial (Low risk) (84) allocated 120 pSS patients to either verum acupuncture or sham acupuncture groups. Statistical analysis following an 8-week intervention and a subsequent 16-week follow-up revealed no significant intergroup differences in the improvement of core symptoms such as xerostomia, xerophthalmia, pain, and fatigue. This outcome prompts consideration of potential explanations, which may include the use of identical acupoints in both the acupuncture and sham acupuncture groups, where different types of stimulation might produce comparable physiological responses, consequently minimizing the observed efficacy gap. Additionally, patients’ psychological expectations should also be considered as interfering factors. This also reflects that in the current research, there is a lack of a recognized and unified standardized operation system for sham acupuncture intervention. There is no unified standard in key aspects such as acupoint selection, needle insertion depth, acupuncture manipulation, and stimulation duration. The operation is not standardized. Moreover, the design concept and selection of the control group in randomized controlled trials have certain limitations, making it difficult to completely eliminate the interference of placebo effect and confounding factors. Conducting systematic research on the sham acupuncture control scheme to fill the shortcomings of the current trial design and further improve the standardization and rigor of acupuncture clinical research is one of the key research directions in the future.

Analysis of therapeutic outcomes consistently demonstrated that the Western medicine monotherapy cohort exhibited the lowest effectiveness rate among all interventional groups. This comparative deficit was observed relative to combination therapy groups receiving either acupuncture with Chinese herbal medicine, acupuncture with Western pharmaceuticals, or the integrated triple-modality approach. It can thus be seen that traditional Chinese medicine has certain advantages and promising prospects in the treatment of Sjögren’s syndrome (SS). Nevertheless, as discussed above, numerous deficiencies still exist in current research regarding its mechanisms of action and clinical efficacy. Accordingly, several prospects for future research are proposed as follows:

① Standardize clinical trials of acupuncture for SS, including optimizing control design for experimental groups, establishing unified efficacy evaluation systems, completing clinical trial registration, and obtaining ethical approval.

② Large-scale, multicenter, rigorously blinded clinical trials should be carried out to produce more definitive and robust evidence for the relative advantages of acupuncture in the management of Sjögren’s syndrome.

③ Systematically investigate the physiological and biochemical mechanisms of acupuncture in treating SS, with further focus on key targets such as the cholinergic anti-inflammatory pathway, NF-κB inflammatory signaling axis, T and B cell immune differentiation, innate immune responses, and intestinal microbiota.

④ Strengthen systematic follow-up and documentation of patients’ long-term prognosis and adverse events, improve the evaluation system for safety and efficacy, and provide more comprehensive evidence-based support for acupuncture therapy for SS.

## 7 Conclusion

Collectively, available evidence indicates that acupuncture, a characteristic external therapy of traditional Chinese medicine, delivers certain clinical advantages in the intervention of Sjögren’s syndrome. Nevertheless, prominent deficiencies persist in relevant research. Mechanistically, acupuncture can modulate pro-inflammatory factors, immunoglobulins and the Th17/Treg balance, yet its complete functional pathways remain unclear. In addition, there is insufficient evidence from animal experiments and clinical trials regarding acupuncture’s regulation of innate immunity and the gut-brain axis. Clinically, existing trials are generally flawed by small sample sizes, lack of blinding designs, and low overall methodological quality. Future research may optimize trial protocols and basic investigations targeting the above limitations, supplement high-quality clinical and preclinical data, and furnish more sufficient and credible evidence for acupuncture therapy in Sjögren’s syndrome.

## References

[1] Fox RI . Sjögren's syndrome. Lancet. (2005) 366:321–31. doi: 10.1016/s0140-6736(05)66990-5 16039337

[2] Both T Dalm VA van Hagen PM van Daele PL . Reviewing primary Sjögren's syndrome: Beyond the dryness - from pathophysiology to diagnosis and treatment. Int J Med Sci. (2017) 14:191–200. doi: 10.7150/ijms.17718 28367079 PMC5370281

[3] Quan J Xinyao Z Xiaopo T Juan J Xun G Rheumatology Branch CAoCM . Guidelines for the diagnosis and treatment of Sjögren's syndrome based on syndrome differentiation and disease combination. J Traditional Chin Med. (2024) 65(4):434–44. doi: 10.13288/j.11-2166/r.2024.04.017

[4] Zhang NZ Shi CS Yao QP Pan GX Wang LL Wen ZX . Prevalence of primary Sjögren's syndrome in China. J Rheumatol. (1995) 22:659–61. 7791159

[5] Ramos-Casals M Brito-Zerón P Bombardieri S Bootsma H De Vita S Dörner T . Eular recommendations for the management of Sjögren's syndrome with topical and systemic therapies. Ann Rheum Dis. (2020) 79:3–18. doi: 10.1136/annrheumdis-2019-216114 31672775

[6] Baer AN Gottenberg JE St Clair EW Sumida T Takeuchi T Seror R . Efficacy and safety of abatacept in active primary Sjögren's syndrome: Results of a phase iii, randomised, placebo-controlled trial. Ann Rheum Dis. (2021) 80:339–48. doi: 10.1136/annrheumdis-2020-218599 33168545 PMC7892395

[7] Wang SQ Zhang LW Wei P Hua H . Is hydroxychloroquine effective in treating primary Sjogren's syndrome: A systematic review and meta-analysis. BMC Musculoskelet Disord. (2017) 18:186. doi: 10.1186/s12891-017-1543-z 28499370 PMC5427554

[8] Zhan Q Zhang J Lin Y Chen W Fan X Zhang D . Pathogenesis and treatment of Sjogren's syndrome: Review and update. Front Immunol. (2023) 14:1127417. doi: 10.3389/fimmu.2023.1127417 36817420 PMC9932901

[9] Ritter J Chen Y Stefanski AL Dörner T . Current and future treatment in primary Sjögren's syndrome - a still challenging development. Joint Bone Spine. (2022) 89:105406. doi: 10.1016/j.jbspin.2022.105406 35537697

[10] List T Lundeberg T Lundström I Lindström F Ravald N . The effect of acupuncture in the treatment of patients with primary Sjögren's syndrome. A controlled study. Acta Odontol Scand. (1998) 56:95–9. doi: 10.1080/00016359850136058 9669460

[11] Furness S Bryan G McMillan R Birchenough S Worthington HV . Interventions for the management of dry mouth: Non-pharmacological interventions. Cochrane Database Syst Rev. (2013) 2013:Cd009603. doi: 10.1002/14651858.CD009603.pub3 24006231 PMC7100870

[12] Kohriyama K Katayama Y . Disproportion of helper T cell subsets in peripheral blood of patients with primary Sjögren's syndrome. Autoimmunity. (2000) 32:67–72. doi: 10.3109/08916930008995989 10958177

[13] Moutsopoulos NM Katsifis GE Angelov N Leakan RA Sankar V Pillemer S . Lack of efficacy of etanercept in Sjögren syndrome correlates with failed suppression of tumour necrosis factor alpha and systemic immune activation. Ann Rheum Dis. (2008) 67:1437–43. doi: 10.1136/ard.2007.077891 18198195

[14] Nordmark G Kristjansdottir G Theander E Appel S Eriksson P Vasaitis L . Association of Ebf1, Fam167a(C8orf13)-Blk and Tnfsf4 gene variants with primary Sjögren's syndrome. Genes Immun. (2011) 12:100–9. doi: 10.1038/gene.2010.44 20861858

[15] Cruz-Tapias P Rojas-Villarraga A Maier-Moore S Anaya JM . Hla and Sjögren's syndrome susceptibility. A meta-analysis of worldwide studies. Autoimmun Rev. (2012) 11:281–7. doi: 10.1016/j.autrev.2011.10.002 22001416

[16] Otsuka K Sato M Tsunematsu T Ishimaru N . Virus infections play crucial roles in the pathogenesis of Sjögren's syndrome. Viruses. (2022) 14(7):1474. doi: 10.3390/v14071474 35891453 PMC9320594

[17] Mandl T Marsal J Olsson P Ohlsson B Andréasson K . Severe intestinal dysbiosis is prevalent in primary Sjögren's syndrome and is associated with systemic disease activity. Arthritis Res Ther. (2017) 19:237. doi: 10.1186/s13075-017-1446-2 29065905 PMC5655865

[18] van der Meulen TA Harmsen HJM Vila AV Kurilshikov A Liefers SC Zhernakova A . Shared gut, but distinct oral microbiota composition in primary Sjögren's syndrome and systemic lupus erythematosus. J Autoimmun. (2019) 97:77–87. doi: 10.1016/j.jaut.2018.10.009 30416033

[19] Verstappen GM Pringle S Bootsma H Kroese FGM . Epithelial-immune cell interplay in primary Sjögren syndrome salivary gland pathogenesis. Nat Rev Rheumatol. (2021) 17:333–48. doi: 10.1038/s41584-021-00605-2 33911236 PMC8081003

[20] Chatzis L Vlachoyiannopoulos PG Tzioufas AG Goules AV . New frontiers in precision medicine for Sjogren's syndrome. Expert Rev Clin Immunol. (2021) 17:127–41. doi: 10.1080/1744666x.2021.1879641 33478279

[21] Vitali C Moutsopoulos HM Bombardieri S . The European Community Study Group on Diagnostic Criteria for Sjögren's Syndrome. Sensitivity and specificity of tests for ocular and oral involvement in Sjögren's syndrome. Ann Rheum Dis. (1994) 53:637–47. doi: 10.1136/ard.53.10.637 7979575 PMC1005429

[22] Chisholm DM Mason DK . Labial salivary gland biopsy in Sjögren's disease. J Clin Pathol. (1968) 21:656–60. doi: 10.1136/jcp.21.5.656 5697370 PMC473887

[23] Christodoulou MI Kapsogeorgou EK Moutsopoulos HM . Characteristics of the minor salivary gland infiltrates in Sjögren's syndrome. J Autoimmun. (2010) 34:400–7. doi: 10.1016/j.jaut.2009.10.004 19889514

[24] Desvaux E Hemon P Soret P Le Dantec C Chatzis L Cornec D . High-content multimodal analysis supports the Il-7/Il-7 receptor axis as a relevant therapeutic target in primary Sjögren's syndrome. J Autoimmun. (2024) 149:103147. doi: 10.1016/j.jaut.2023.103147 38114349

[25] Rivière E Pascaud J Tchitchek N Boudaoud S Paoletti A Ly B . Salivary gland epithelial cells from patients with Sjögren's syndrome induce B-lymphocyte survival and activation. Ann Rheum Dis. (2020) 79:1468–77. doi: 10.1136/annrheumdis-2019-216588 32843324

[26] Carvajal P Aguilera S Jara D Indo S Barrera MJ González S . Hsa-mir-424-5p and hsa-mir-513c-3p dysregulation mediated by Ifn-Γ is associated with salivary gland dysfunction in Sjögren's syndrome patients. J Autoimmun. (2023) 138:103037. doi: 10.1016/j.jaut.2023.103037 37229808

[27] Hinrichs AC Kruize AA Leavis HL van Roon JAG . In patients with primary Sjögren's syndrome innate-like Mait cells display upregulated Il-7r, Ifn-Γ, and Il-21 expression and have increased proportions of Ccr9 and Cxcr5-expressing cells. Front Immunol. (2022) 13:1017157. doi: 10.3389/fimmu.2022.1017157 36505431 PMC9729251

[28] Cao T Zhou J Liu Q Mao T Chen B Wu Q . Interferon-Γ induces salivary gland epithelial cell ferroptosis in Sjogren's syndrome via Jak/Stat1-mediated inhibition of System Xc(). Free Radic Biol Med. (2023) 205:116–28. doi: 10.1016/j.freeradbiomed.2023.05.027 37286044

[29] Rivière E Pascaud J Virone A Dupré A Ly B Paoletti A . Interleukin-7/interferon axis drives T cell and salivary gland epithelial cell interactions in Sjögren's syndrome. Arthritis Rheumatol. (2021) 73:631–40. doi: 10.1002/art.41558 33058491

[30] Pène J Gauchat JF Lécart S Drouet E Guglielmi P Boulay V . Cutting edge: Il-21 is a switch factor for the production of Igg1 and Igg3 by human B cells. J Immunol. (2004) 172:5154–7. doi: 10.4049/jimmunol.172.9.5154 15100251

[31] Kwok SK Lee J Yu D Kang KY Cho ML Kim HR . A pathogenetic role for Il-21 in primary Sjögren syndrome. Nat Rev Rheumatol. (2015) 11:368–74. doi: 10.1038/nrrheum.2014.225 25584898

[32] Kang KY Kim HO Kwok SK Ju JH Park KS Sun DI . Impact of interleukin-21 in the pathogenesis of primary Sjögren's syndrome: Increased serum levels of interleukin-21 and its expression in the labial salivary glands. Arthritis Res Ther. (2011) 13:R179. doi: 10.1186/ar3504 22030011 PMC3308114

[33] Zhou J Pathak JL Cao T Chen B Wei W Hu S . Cd4 T cell-secreted Ifn-Γ in Sjögren's syndrome induces salivary gland epithelial cell ferroptosis. Biochim Biophys Acta Mol Basis Dis. (2024) 1870:167121. doi: 10.1016/j.bbadis.2024.167121 38471652

[34] Kamada N Seo SU Chen GY Núñez G . Role of the gut microbiota in immunity and inflammatory disease. Nat Rev Immunol. (2013) 13:321–35. doi: 10.1038/nri3430 23618829

[35] Lynch SV Pedersen O . The human intestinal microbiome in health and disease. N Engl J Med. (2016) 375:2369–79. doi: 10.1056/NEJMra1600266 27974040

[36] Schluter J Peled JU Taylor BP Markey KA Smith M Taur Y . The gut microbiota is associated with immune cell dynamics in humans. Nature. (2020) 588:303–7. doi: 10.1038/s41586-020-2971-8 33239790 PMC7725892

[37] Koboziev I Reinoso Webb C Furr KL Grisham MB . Role of the enteric microbiota in intestinal homeostasis and inflammation. Free Radic Biol Med. (2014) 68:122–33. doi: 10.1016/j.freeradbiomed.2013.11.008 24275541 PMC3943931

[38] Zhong D Wu C Zeng X Wang Q . The role of gut microbiota in the pathogenesis of rheumatic diseases. Clin Rheumatol. (2018) 37:25–34. doi: 10.1007/s10067-017-3821-4 28914372

[39] Moon J Choi SH Yoon CH Kim MK . Gut dysbiosis is prevailing in Sjögren's syndrome and is related to dry eye severity. PloS One. (2020) 15:e0229029. doi: 10.1371/journal.pone.0229029 32059038 PMC7021297

[40] Li Y Li Z Sun W Wang M Li M . Characteristics of gut microbiota in patients with primary Sjögren's syndrome in Northern China. PloS One. (2022) 17:e0277270. doi: 10.1371/journal.pone.0277270 36355832 PMC9648750

[41] Wang F Zhufeng Y Chen Z Xu J Cheng Y . The composition and function profile of the gut microbiota of patients with primary Sjögren's syndrome. Clin Rheumatol. (2023) 42:1315–26. doi: 10.1007/s10067-022-06451-1 36598587

[42] Rusthen S Kristoffersen AK Young A Galtung HK Petrovski B Palm Ø . Dysbiotic salivary microbiota in dry mouth and primary Sjögren's syndrome patients. PloS One. (2019) 14:e0218319. doi: 10.1371/journal.pone.0218319 31211815 PMC6581286

[43] Cano-Ortiz A Laborda-Illanes A Plaza-Andrades I Membrillo Del Pozo A Villarrubia Cuadrado A Rodríguez Calvo de Mora M . Connection between the gut microbiome, systemic inflammation, gut permeability and Foxp3 expression in patients with primary Sjögren's syndrome. Int J Mol Sci. (2020) 21(22):8733. doi: 10.3390/ijms21228733 33228011 PMC7699261

[44] Bombardieri M Argyropoulou OD Ferro F Coleby R Pontarini E Governato G . One year in review 2020: Pathogenesis of primary Sjögren's syndrome. Clin Exp Rheumatol. (2020) 38 Suppl 126:3–9. 33025887

[45] Jin L Dai M Li C Wang J Wu B . Risk factors for primary Sjögren's syndrome: A systematic review and meta-analysis. Clin Rheumatol. (2023) 42:327–38. doi: 10.1007/s10067-022-06474-8 36534351 PMC9873717

[46] Yao Q Song Z Wang B Qin Q Zhang JA . Identifying key genes and functionally enriched pathways in Sjögren's syndrome by weighted gene co-expression network analysis. Front Genet. (2019) 10:1142. doi: 10.3389/fgene.2019.01142 31798636 PMC6863930

[47] Tincani A Andreoli L Cavazzana I Doria A Favero M Fenini MG . Novel aspects of Sjögren's syndrome in 2012. BMC Med. (2013) 11:93. doi: 10.1186/1741-7015-11-93 23556533 PMC3616867

[48] García-Carrasco M Ramos-Casals M Rosas J Pallarés L Calvo-Alen J Cervera R . Primary Sjögren syndrome: Clinical and immunologic disease patterns in a cohort of 400 patients. Med (Baltimore). (2002) 81:270–80. doi: 10.1097/00005792-200207000-00003 12169882

[49] ter Borg EJ Risselada AP Kelder JC . Relation of systemic autoantibodies to the number of extraglandular manifestations in primary Sjögren's syndrome: A retrospective analysis of 65 patients in the Netherlands. Semin Arthritis Rheum. (2011) 40:547–51. doi: 10.1016/j.semarthrit.2010.07.006 20864144

[50] Seror R Bootsma H Bowman SJ Dörner T Gottenberg JE Mariette X . Outcome measures for primary Sjögren's syndrome. J Autoimmun. (2012) 39:97–102. doi: 10.1016/j.jaut.2012.01.013 22365784

[51] Zhou X Sun Z Cui Y Yin H . Novel insights of acupuncture in ischemic stroke: Orchestrating neuro-endocrine-immune network. Front Immunol. (2026) 17:1772371. doi: 10.3389/fimmu.2026.1772371 41939880 PMC13043432

[52] Ding N Wei Q Deng W Sun X Zhang J Gao W . Electroacupuncture alleviates inflammation of dry eye diseases by regulating the A7nachr/Nf-Kb signaling pathway. Oxid Med Cell Longev. (2021) 2021:6673610. doi: 10.1155/2021/6673610 33897942 PMC8052151

[53] Kim EY Moudgil KD . Regulation of autoimmune inflammation by pro-inflammatory cytokines. Immunol Lett. (2008) 120:1–5. doi: 10.1016/j.imlet.2008.07.008 18694783 PMC2577081

[54] Kang EH Lee YJ Hyon JY Yun PY Song YW . Salivary cytokine profiles in primary Sjögren's syndrome differ from those in non-Sjögren sicca in terms of Tnf-A levels and Th-1/Th-2 ratios. Clin Exp Rheumatol. (2011) 29:970–6. 22132900

[55] Limaye A Hall BE Zhang L Cho A Prochazkova M Zheng C . Targeted Tnf-A overexpression drives salivary gland inflammation. J Dent Res. (2019) 98:713–9. doi: 10.1177/0022034519837240 30958728 PMC6535923

[56] Ciccia F Guggino G Rizzo A Ferrante A Raimondo S Giardina A . Potential involvement of Il-22 and Il-22-producing cells in the inflamed salivary glands of patients with Sjogren's syndrome. Ann Rheum Dis. (2012) 71:295–301. doi: 10.1136/ard.2011.154013 21979002

[57] Lin X Rui K Deng J Tian J Wang X Wang S . Th17 cells play a critical role in the development of experimental Sjögren's syndrome. Ann Rheum Dis. (2015) 74:1302–10. doi: 10.1136/annrheumdis-2013-204584 24573745

[58] Nguyen CQ Yin H Lee BH Carcamo WC Chiorini JA Peck AB . Pathogenic effect of interleukin-17a in induction of Sjögren's syndrome-like disease using adenovirus-mediated gene transfer. Arthritis Res Ther. (2010) 12:R220. doi: 10.1186/ar3207 21182786 PMC3046533

[59] Roescher N Tak PP Illei GG . Cytokines in Sjogren's syndrome: Potential therapeutic targets. Ann Rheum Dis. (2010) 69:945–8. doi: 10.1136/ard.2009.115378 20410069 PMC3044243

[60] Youinou P Pers JO . Disturbance of cytokine networks in Sjögren's syndrome. Arthritis Res Ther. (2011) 13:227. doi: 10.1186/ar3348 21745420 PMC3239335

[61] Kim J Kim JG Li Y You S Lee N Kim WU . Lsp1 deficiency increases Il-17-expressing T cells and accelerates primary Sjögren's syndrome. Clin Immunol. (2025) 280:110548. doi: 10.1016/j.clim.2025.110548 40588087

[62] Hwang SH Woo JS Moon J Yang S Park JS Lee J . Il-17 and Ccr9(+)A4β7(-) Th17 cells promote salivary gland inflammation, dysfunction, and cell death in Sjögren's syndrome. Front Immunol. (2021) 12:721453. doi: 10.3389/fimmu.2021.721453 34539657 PMC8440850

[63] Azuma M Motegi K Aota K Hayashi Y Sato M . Role of cytokines in the destruction of acinar structure in Sjögren's syndrome salivary glands. Lab Invest. (1997) 77:269–80. 9314950

[64] Willeke P Schotte H Schlüter B Erren M Becker H Dyong A . Interleukin 1beta and tumour necrosis factor alpha secreting cells are increased in the peripheral blood of patients with primary Sjögren's syndrome. Ann Rheum Dis. (2003) 62:359–62. doi: 10.1136/ard.62.4.359 12634239 PMC1754485

[65] Tracey KJ . The inflammatory reflex. Nature. (2002) 420:853–9. doi: 10.1038/nature01321 12490958

[66] Borovikova LV Ivanova S Zhang M Yang H Botchkina GI Watkins LR . Vagus nerve stimulation attenuates the systemic inflammatory response to endotoxin. Nature. (2000) 405:458–62. doi: 10.1038/35013070 10839541

[67] Borovikova LV Ivanova S Nardi D Zhang M Yang H Ombrellino M . Role of vagus nerve signaling in Cni-1493-mediated suppression of acute inflammation. Auton Neurosci. (2000) 85:141–7. doi: 10.1016/s1566-0702(00)00233-2 11189021

[68] Bernik TR Friedman SG Ochani M DiRaimo R Ulloa L Yang H . Pharmacological stimulation of the cholinergic antiinflammatory pathway. J Exp Med. (2002) 195:781–8. doi: 10.1084/jem.20011714 11901203 PMC2193742

[69] Wang H Yu M Ochani M Amella CA Tanovic M Susarla S . Nicotinic acetylcholine receptor alpha7 subunit is an essential regulator of inflammation. Nature. (2003) 421:384–8. doi: 10.1038/nature01339 12508119

[70] Tracey KJ . Physiology and immunology of the cholinergic antiinflammatory pathway. J Clin Invest. (2007) 117:289–96. doi: 10.1172/jci30555 17273548 PMC1783813

[71] Tracey KJ . Reflex control of immunity. Nat Rev Immunol. (2009) 9:418–28. doi: 10.1038/nri2566 19461672 PMC4535331

[72] Rosas-Ballina M Tracey KJ . Cholinergic control of inflammation. J Intern Med. (2009) 265:663–79. doi: 10.1111/j.1365-2796.2009.02098.x 19493060 PMC4540232

[73] Kwok SK Cho ML Her YM Oh HJ Park MK Lee SY . Tlr2 ligation induces the production of Il-23/Il-17 via Il-6, Stat3 and Nf-Kb pathway in patients with primary Sjogren's syndrome. Arthritis Res Ther. (2012) 14:R64. doi: 10.1186/ar3780 22417709 PMC3446432

[74] Tian MH Zhang YN Sun WF Liu H Tang Y . Acupuncture at "Die E acupoint" alleviates inflammatory reaction via inhibiting Tlr4/Myd88/Nf-Kb signaling in rats with allergic rhinitis. Zhen Ci Yan Jiu. (2024) 49:456–62. doi: 10.13702/j.1000-0607.20230724 38764116

[75] Zhe M Lingjie L Huangan W Jianhua Z Yaying L Yu Q . Effect of moxibustion on P2x7r/Nf-Kb pathway in ulcerative colitis rats with repeated induction of Dss. Acupuncture Herbal Med. (2025) 5:205–16. doi: 10.1097/hm9.0000000000000159 42348292

[76] Liu R Zhang Y Li K Xu H Cheng Z Pang F . Effect of acupuncture on regulating Il-17, Tnf-α and Aqps in Sjögren's syndrome. Oral Dis. (2024) 30:50–62. doi: 10.1111/odi.14680 37518974

[77] Schroeder HW Jr. Cavacini L . Structure and function of immunoglobulins. J Allergy Clin Immunol. (2010) 125:S41–52. doi: 10.1016/j.jaci.2009.09.046 20176268 PMC3670108

[78] Talal N Asofsky R Lightbody P . Immunoglobulin synthesis by salivary gland lymphoid cells in Sjögren's syndrome. J Clin Invest. (1970) 49:49–54. doi: 10.1172/jci106221 4188268 PMC322443

[79] Jiang Q Zhang H Pang R Chen J Liu Z Zhou X . Acupuncture for primary Sjögren syndrome (Pss) on symptomatic improvements: Study protocol for a randomized controlled trial. BMC Complement Altern Med. (2017) 17:61. doi: 10.1186/s12906-017-1559-9 28103850 PMC5248458

[80] Haldorsen K Bjelland I Bolstad AI Jonsson R Brun JG . A five-year prospective study of fatigue in primary Sjögren's syndrome. Arthritis Res Ther. (2011) 13:R167. doi: 10.1186/ar3487 21996338 PMC3308101

[81] Tsukamoto M Suzuki K Takeuchi T . Ten-year observation of patients with primary Sjögren's syndrome: Initial presenting characteristics and the associated outcomes. Int J Rheum Dis. (2019) 22:929–33. doi: 10.1111/1756-185x.13464 30588773

[82] Liu F Wang Y Lyu K Du X Zhou M Shi J . Acupuncture and its ability to restore and maintain immune homeostasis. Qjm. (2024) 117:167–76. doi: 10.1093/qjmed/hcad134 37318994

[83] Xiao QS Ma MY Zhang XS Deng MH Yang Yan Z . Effect of acupuncture on prognosis and immune function of sepsis patients. Zhongguo Zhong Xi Yi Jie He Za Zhi. (2015) 35:783–6. doi: 10.7661/CJIM.2015.07.0783 26380438

[84] Zhou X Xu H Chen J Wu H Zhang Y Tian F . Efficacy and safety of acupuncture on symptomatic improvement in primary Sjögren's syndrome: A randomized controlled trial. Front Med (Lausanne). (2022) 9:878218. doi: 10.3389/fmed.2022.878218 35602489 PMC9121854

[85] Ulloa L Quiroz-Gonzalez S Torres-Rosas R . Nerve stimulation: Immunomodulation and control of inflammation. Trends Mol Med. (2017) 23:1103–20. doi: 10.1016/j.molmed.2017.10.006 29162418 PMC5724790

[86] Zhang R Lao L Ren K Berman BM . Mechanisms of acupuncture-electroacupuncture on persistent pain. Anesthesiology. (2014) 120:482–503. doi: 10.1097/aln.0000000000000101 24322588 PMC3947586

[87] Liu W Liu B Zheng HX . Observation on therapeutic effect of acupuncture and moxibustion in 60 cases of Sjogren syndrome. Zhongguo Zhen Jiu. (2005) 25:101–2. doi: 10.3321/j.issn:0255-2930.2005.02.010 16312891

[88] Hua B Peng Y . Clinical observation of electroacupuncture in treating Sjögren's syndrome and its impact on immune function. J Clin Acupuncture Moxibustion. (2009) 25:9–11. doi: 10.3969/j.issn.1005-0779.2009.07.004

[89] Yao Y Ma JF Chang C Xu T Gao CY Gershwin ME . Immunobiology of T cells in Sjögren's syndrome. Clin Rev Allergy Immunol. (2021) 60:111–31. doi: 10.1007/s12016-020-08793-7 32390096

[90] Luckheeram RV Zhou R Verma AD Xia B . Cd4^+^T cells: Differentiation and functions. Clin Dev Immunol. (2012) 2012:925135. doi: 10.1155/2012/925135 22474485 PMC3312336

[91] Verstappen GM Corneth OBJ Bootsma H Kroese FGM . Th17 cells in primary Sjögren's syndrome: Pathogenicity and plasticity. J Autoimmun. (2018) 87:16–25. doi: 10.1016/j.jaut.2017.11.003 29191572

[92] Liston A Gray DH . Homeostatic control of regulatory T cell diversity. Nat Rev Immunol. (2014) 14:154–65. doi: 10.1038/nri3605 24481337

[93] Rosenblum MD Way SS Abbas AK . Regulatory T cell memory. Nat Rev Immunol. (2016) 16:90–101. doi: 10.1038/nri.2015.1 26688349 PMC5113825

[94] Christodoulou MI Kapsogeorgou EK Moutsopoulos NM Moutsopoulos HM . Foxp3+ T-regulatory cells in Sjogren's syndrome: Correlation with the grade of the autoimmune lesion and certain adverse prognostic factors. Am J Pathol. (2008) 173:1389–96. doi: 10.2353/ajpath.2008.080246 18818377 PMC2570129

[95] Alunno A Carubbi F Bistoni O Caterbi S Bartoloni E Mirabelli G . T regulatory and T helper 17 cells in primary Sjögren's syndrome: Facts and perspectives. Mediators Inflammation. (2015) 2015:243723. doi: 10.1155/2015/243723 26060357 PMC4427804

[96] Miao M Hao Z Guo Y Zhang X Zhang S Luo J . Short-term and low-dose Il-2 therapy restores the Th17/Treg balance in the peripheral blood of patients with primary Sjögren's syndrome. Ann Rheum Dis. (2018) 77:1838–40. doi: 10.1136/annrheumdis-2018-213036 29936436

[97] Li B Xing Y Gan Y He J Hua H . Labial gland-derived mesenchymal stem cells and their exosomes ameliorate murine Sjögren's syndrome by modulating the balance of Treg and Th17 cells. Stem Cell Res Ther. (2021) 12:478. doi: 10.1186/s13287-021-02541-0 34446113 PMC8390194

[98] Hao LR Li XF Gao C Cao L Han ZY Gao H . Th17/Treg cell level and clinical characteristics of peripheral blood of patients with Sjogren's syndrome complicated with primary biliary cirrhosis. Med (Baltimore). (2019) 98:e15952. doi: 10.1097/md.0000000000015952 31192933 PMC6587605

[99] Ivanov II McKenzie BS Zhou L Tadokoro CE Lepelley A Lafaille JJ . The orphan nuclear receptor Rorgammat directs the differentiation program of proinflammatory Il-17+ T helper cells. Cell. (2006) 126:1121–33. doi: 10.1016/j.cell.2006.07.035 16990136

[100] Ichiyama K Yoshida H Wakabayashi Y Chinen T Saeki K Nakaya M . Foxp3 inhibits Rorgammat-mediated Il-17a Mrna transcription through direct interaction with Rorgammat. J Biol Chem. (2008) 283:17003–8. doi: 10.1074/jbc.M801286200 18434325

[101] Zhou L Lopes JE Chong MM Ivanov II Min R Victora GD . Tgf-beta-induced Foxp3 inhibits T(H)17 cell differentiation by antagonizing Rorgammat function. Nature. (2008) 453:236–40. doi: 10.1038/nature06878 18368049 PMC2597437

[102] Wang Y Zhang L Li L Hu H Pan P Zhang B . Electroacupuncture improves blood pressure in Shrs by regulating the immune balance between Th17 and Treg. Evid Based Complement Alternat Med. (2020) 2020:5375981. doi: 10.1155/2020/5375981 32724324 PMC7366219

[103] Xinhua L Xiaoli Z Chao W Meiqing S . Effect of acupuncture combined with hydroxychloroquine sulfate on the ratio of T helper cell 17/regulatory T cells in patients with primary Sjogren′s syndrome. China Modern Med. (2024) 31:106–10. doi: 10.3969/j.issn.1674-4721.2024.02.026

[104] Shimizu T Nakamura H Kawakami A . Role of the innate immunity signaling pathway in the pathogenesis of Sjögren's syndrome. Int J Mol Sci. (2021) 22(6):3090. doi: 10.3390/ijms22063090 33803026 PMC8002742

[105] Medzhitov R Janeway C Jr . Innate immunity. N Engl J Med. (2000) 343:338–44. doi: 10.1056/nejm200008033430506 10922424

[106] Medzhitov R Janeway CA Jr . Innate immunity: The virtues of a nonclonal system of recognition. Cell. (1997) 91:295–8. doi: 10.1016/s0092-8674(00)80412-2 9363937

[107] Medzhitov R . Pattern recognition theory and the launch of modern innate immunity. J Immunol. (2013) 191:4473–4. doi: 10.4049/jimmunol.1302427 24141853

[108] Gilliet M Cao W Liu YJ . Plasmacytoid dendritic cells: Sensing nucleic acids in viral infection and autoimmune diseases. Nat Rev Immunol. (2008) 8:594–606. doi: 10.1038/nri2358 18641647

[109] Kawai T Akira S . The role of pattern-recognition receptors in innate immunity: Update on toll-like receptors. Nat Immunol. (2010) 11:373–84. doi: 10.1038/ni.1863 20404851

[110] Spachidou MP Bourazopoulou E Maratheftis CI Kapsogeorgou EK Moutsopoulos HM Tzioufas AG . Expression of functional toll-like receptors by salivary gland epithelial cells: Increased Mrna expression in cells derived from patients with primary Sjögren's syndrome. Clin Exp Immunol. (2007) 147:497–503. doi: 10.1111/j.1365-2249.2006.03311.x 17302899 PMC1810489

[111] Jabri B Abadie V . Il-15 functions as a danger signal to regulate tissue-resident T cells and tissue destruction. Nat Rev Immunol. (2015) 15:771–83. doi: 10.1038/nri3919 26567920 PMC5079184

[112] Patidar M Yadav N Dalai SK . Interleukin 15: A key cytokine for immunotherapy. Cytokine Growth Factor Rev. (2016) 31:49–59. doi: 10.1016/j.cytogfr.2016.06.001 27325459

[113] Barrera MJ Aguilera S Veerman E Quest AF Díaz-Jiménez D Urzúa U . Salivary mucins induce a toll-like receptor 4-mediated pro-inflammatory response in human submandibular salivary cells: Are mucins involved in Sjögren's syndrome? Rheumatol (Oxford). (2015) 54:1518–27. doi: 10.1093/rheumatology/kev026 25802401

[114] Banchereau J Steinman RM . Dendritic cells and the control of immunity. Nature. (1998) 392:245–52. doi: 10.1038/32588 9521319

[115] Akira S Takeda K Kaisho T . Toll-like receptors: Critical proteins linking innate and acquired immunity. Nat Immunol. (2001) 2:675–80. doi: 10.1038/90609 11477402

[116] Medzhitov R . Toll-like receptors and innate immunity. Nat Rev Immunol. (2001) 1:135–45. doi: 10.1038/35100529 11905821

[117] McCoy CE O'Neill LA . The role of toll-like receptors in macrophages. Front Biosci. (2008) 13:62–70. doi: 10.2741/2660 17981528

[118] Aderem A . Phagocytosis and the inflammatory response. J Infect Dis. (2003) 187 Suppl 2:S340–5. doi: 10.1086/374747 12792849

[119] Underhill DM Gantner B . Integration of toll-like receptor and phagocytic signaling for tailored immunity. Microbes Infect. (2004) 6:1368–73. doi: 10.1016/j.micinf.2004.08.016 15596122

[120] Blander JM Medzhitov R . Toll-dependent selection of microbial antigens for presentation by dendritic cells. Nature. (2006) 440:808–12. doi: 10.1038/nature04596 16489357

[121] Medzhitov R Preston-Hurlburt P Janeway CA Jr . A human homologue of the Drosophila Toll protein signals activation of adaptive immunity. Nature. (1997) 388:394–7. doi: 10.1038/41131 9237759

[122] Wu X Ding N Liang S Han Y Fan S Wu Y . Electroacupuncture improves scopolamine hydrobromide induced dry eye in mice via inhibiting ocular surface inflammation and regulating the Hmgb1-related signaling pathways. Front Med (Lausanne). (2025) 12:1664376. doi: 10.3389/fmed.2025.1664376 41210848 PMC12589004

[123] Chen T Xiong Y Long M Zheng D Ke H Xie J . Electro-acupuncture pretreatment at Zusanli (St36) acupoint attenuates lipopolysaccharide-induced inflammation in rats by inhibiting Ca(2+) influx associated with cannabinoid Cb2 receptors. Inflammation. (2019) 42:211–20. doi: 10.1007/s10753-018-0885-5 30168040

[124] Jiang Z Liu L Zhang L Li L Ding B Hu L . Effects of "Brain-gut coherence" method of acupuncture on motor function and intestinal microflora in the patients with cerebral ischemic stroke. Zhongguo Zhen Jiu. (2024) 44:740–8. doi: 10.13703/j.0255-2930.20231109-k0009 38986585

[125] Xie J Li J Sun Q Jiang J . Clinical efficacy of mind-regulating acupuncture on post-stroke depression based on the "Microbiota-gut-brain axis" theory: A randomized controlled study. Neuropsychiatr Dis Treat. (2025) 21:1349–58. doi: 10.2147/ndt.S525238 40656933 PMC12254467

[126] Li S Wang S Li S Liu C Sun J Cao J . Brain-gut-microbiota axis: An effective target of abdominal acupuncture therapy for post-stroke functional constipation. Complement Ther Med. (2025) 95:103286. doi: 10.1016/j.ctim.2025.103286 41176186

[127] Xianjun X Peiwen X Haiyan Q Di Q Lu W Rongjiang J . Effects of electroacupuncture on gut microbiota in a rat model of urticaria. Acupuncture Herbal Med. (2025) 5:103–14. doi: 10.1097/hm9.0000000000000145 42348292

[128] Ma XP Hong J An CP Zhang D Huang Y Wu HG . Acupuncture-moxibustion in treating irritable bowel syndrome: How does it work? World J Gastroenterol. (2014) 20:6044–54. doi: 10.3748/wjg.v20.i20.6044 24876727 PMC4033444

[129] Lim B Manheimer E Lao L Ziea E Wisniewski J Liu J . Acupuncture for treatment of irritable bowel syndrome. Cochrane Database Syst Rev. (2006) Cd005111. doi: 10.1002/14651858.CD005111.pub2 17054239

[130] Sun Z Wang X Feng S Xie C Xing Y Guo L . A review of neuroendocrine immune system abnormalities in Ibs based on the brain-gut axis and research progress of acupuncture intervention. Front Neurosci. (2023) 17:934341. doi: 10.3389/fnins.2023.934341 36968497 PMC10034060

[131] Jin Y Hu F Zhu J . Exploration of acupuncture therapy in the treatment of mild cognitive impairment based on the brain-gut axis theory. Front Hum Neurosci. (2022) 16:891411. doi: 10.3389/fnhum.2022.891411 36204718 PMC9531719

[132] Jianbin L Wei L . Systematic review and meta-analysis of the efficacy of acupuncture and acupuncture combined with herbal medicine for treating xerostomia and dry eye in patients with Sjogren’s syndrome. Tianjin J Traditional Chin Med. (2025) 42:999–1010. doi: 10.11656/j.issn.1672-1519.2025.08.10

[133] Zhang SY . The Tcm etiology, pathogenesy and differential treatment for Sjogren's syndrome. J Tradit Chin Med. (2011) 31:73–8. doi: 10.1016/s0254-6272(11)60017-4 21563513

[134] Yi Y Caijiao L Wei W Mingshen L Lanlan Z Xiaorong D . Exploration of acupuncture therapy for Sjögren’s syndrome based on the theory of "Sweat pores, Qi and body fluid. Hunan J Traditional Chin Med. (2025) 41:99–102. doi: 10.16808/j.cnki.issn1003-7705.2025.07.023

[135] Blom M Lundeberg T . Long-term follow-up of patients treated with acupuncture for xerostomia and the influence of additional treatment. Oral Dis. (2000) 6:15–24. doi: 10.1111/j.1601-0825.2000.tb00316.x 10673783

[136] Gomes-Silva JM Torres CP Teixeira LR Saraiva M Oliveira FR Rocha EM . Prospective sham-controlled trial: Acupuncture for symptom-relieving in patients with Sjögren's disease. Clin Rheumatol. (2025) 44:1971–82. doi: 10.1007/s10067-025-07410-2 40178679

[137] Cui Y Xia L Li L Zhao Q Chen S Gu Z . Anxiety and depression in primary Sjögren's syndrome: A cross-sectional study. BMC Psychiatry. (2018) 18:131. doi: 10.1186/s12888-018-1715-x 29769121 PMC5956972

[138] Weiwei Q . Clinical efficacy evaluationof acupuncture in the treatment of primary Sögren's syndrome with anxiety and deprepsson. In: China Academy of Chinese Medical Sciences Master’s thesis, Beijing, China: China Academy of Chinese Medical Sciences, (2019).

[139] Shuang W Hefeng Z Chengwu W . Effect analysis of dryness tongluo acupuncture in the treatment of Sjogren syndrome. Chin J Convalescent Med. (2019) 28:16–8. doi: 10.13517/j.cnki.ccm.2019.01.006

[140] Bin R Min Y . Clinical observation on electroacupuncture for 45 cases of Sjögren’s syndrome. J Sichuan Traditional Chin Med. (2012) 30:116–7.

[141] Xueli Z . Clinical observation on 30 patients with Sjögren’s syndrome treated by acupuncture for moistening dryness and unblocking collaterals. Traditional Chin Medicinal Res. (2013) 26:46–8. doi: 10.3969/j.issn.1001-6910.2013.09.26

[142] Xiaocan C Wei L Xiaojun L Qin W Yanguang F . Observations on the therapeutic effect of electroacupuncture on xerostomia in Sjogren syndrome. Shanghai J Acupuncture Moxibustion. (2014) 33:542–3. doi: 10.13460/j.issn.1005-0957.2014.06.0542

[143] Lan Y . Clinical observation on treatment of primary Sjögren's syndromewith dry eye and dry mouth by Xinwu needle. In: Chengdu University of TCM Master’s thesis, Chengdu, Sichuan, China: Chengdu University of Traditional Chinese Medicine, (2023).

[144] Chao W Li X Chen Y . Clinical observation on treating 26 cases of primary Sjogren’s syndrome of yin deficiency and blood dryness type by acupuncture and medicine. Clin J Chin Med. (2023) 15:82–6. doi: 10.3969/j.issn.1674-7860.2023.16.016

[145] Xu D Guo L . Therapeutic observation on the treatment of Sjogren's syndrome by majorly needling back-shu points. Shanghai J Acupuncture Moxibustion. (2013) 32:28–9. doi: 10.3969/j.issn.1005-0957.2013.01.028

[146] Alunno A Carubbi F Bartoloni E Cipriani P Giacomelli R Gerli R . The kaleidoscope of neurological manifestations in primary Sjögren's syndrome. Clin Exp Rheumatol. (2019) 37 Suppl 118:192–8. 31464676

[147] Chai J Logigian EL . Neurological manifestations of primary Sjogren's syndrome. Curr Opin Neurol. (2010) 23:509–13. doi: 10.1097/WCO.0b013e32833de6ab 20689426

[148] Liampas A Parperis K Erotocritou MF Nteveros A Papadopoulou M Moschovos C . Primary Sjögren syndrome-related peripheral neuropathy: A systematic review and meta-analysis. Eur J Neurol. (2023) 30:255–65. doi: 10.1111/ene.15555 36086910 PMC10087501

[149] Longsheng L Ying S Wenrong W Jinchun C Huirong L . Clinical study on reducling phlegm and promoting blood acupuncture in the treatment of Sjogren’s syndrome with peripheral neuropathy. Chin Med Modern Distance Educ China. (2020) 18:107–10. doi: 10.3969/j.issn.1672-2779.2020.08.045

[150] Ren J Zhang M . Efficacy observation of acupuncture combined with Chinese medicine in the treatment of primary Sjögren's syndrome with liver-kidney yin deficiency type in women. Inner Mongolia J Traditional Chin Med. (2023) 42:45–7. doi: 10.16040/j.cnki.cn15-1101.2023.02.048

[151] Zhiqiang G Wengang H Shaoying Z Zhancheng W Xiuhui Z Fubin J . Clinical observation of guibieziyin powder combined with acupoint acupuncture in the treatment of Sjögren’s syndrome. China Pharm. (2021) 30:74–7. doi: 10.3969/j.issn.1006-4931.2021.06.021

[152] Dake X Lei X Wei H Meimei X Liang G Bo X . Treatment of acupuncture combined with ziyin shengjin prescription: clinical study of 30 cases of yin-deficiency and fluid consumption type of primary Sjgren syndrome. Jiangsu J Traditional Chin Med. (2020) 52:59–62. doi: 10.19844/j.cnki.1672-397X.2020.09.022

[153] Yanguang F Qin W Changhong L Silu W Shujun W Xiaocan C . Clinical study on treatment of Sjogren's syndrome by electroacupuncture combined with wumei pill addition and subtraction. J Clin Pathological Res. (2018) 38:2621–6. doi: 10.3978/j.issn.2095-6959.2018.12.016

[154] Xiaocan W Xiaoying Y . Shashen maidong decoction combined with acupuncture in the treatment of Sjögren´s syndrome. Guangming J Chin Med. (2025) 40:2738–41. doi: 10.3969/j.issn.1003-8914.2025.13.032

[155] Beng L Yuan X Xuemei P Jun Z . Clinical observation on the effects of disease index linked to primary Sjogren syndrome by acupuncture combined with traditional Chinese medicine. Tianjin J Traditional Chin Med. (2017) 34:26–31. doi: 10.11656/j.issn.1672-1519.2017.01.07

[156] Fuyu C Bin X Wei L . Clinical observation of acupuncture and tcm in treatment of 30 cases primary Sjogren syndrome. J Pract Traditional Chin Internal Med. (2012) 26:60–1. doi: 10.3969/j.issn.1671-7813.2012.09.37

[157] Aiping C Bo W Cong M . A clinical observation of 27 cases of primary Sjögren’s syndrome treated with filiform needling and ’yi qi sheng jin san’. Jiangsu J Traditional Chin Med. (2019) 51:64–6. doi: 10.3969/j.issn.1672-397X.2019.10.022

[158] Ge L Zhao R Deng J Zhang H . Strongthened effect of acupuncture on lus' runzao decoction in treatment of dry-eye patients of primary Sjögren's syndrome. China J Traditional Chin Med Pharm. (2017) 32:344–6.

[159] Xiaoshuang H Lin L Fei L Yaping L Dongjie H . Effects of linggui zhugan decoction combined with acupuncture on the improvement of salivary gland, lacrimal gland secretion function, and immune function in patients with Sjögren's syndrome. Chin J Modern Appl Pharm. (2025) 42:454–9. doi: 10.13748/j.cnki.issn1007-7693.20233243

[160] Jianli Z Jinhui W Xinju D Yanyan Z Xiaochao G Xide Z . Efficacy of huayu jiedu decoction combined with acupuncture in the treatment of primary Sjögren′s syndrome and its effects on serum igg, iga and crp. Traditional Chin Medicinal Res. (2023) 36:44–7. doi: 10.3969/j.issn.1001-6910.2023.04.12

[161] Guie X Shufeng W . Clinical effect and safety study of modified gualou qumai decoction combined with acupoint acupuncture in the treatment of Sjögren's syndrome. J Sichuan Traditional Chin Med. (2023) 41:115–8. doi: 10.3969/j.issn.1000-3649.2023.2.sczy202302034

[162] Haiqin G Wenxiao R Qiang X Hongshan Z Linhe M Xiaojun W . Clinical observation of suangan granula combined with acupuncture on the treatment of primary Sjögren's syndrome. Hebei J Traditional Chin Med. (2015) 37:1460–2. doi: 10.3969/j.issn.1002-2619.2015.10.006

[163] Shuhong L . Observation on clinical efficacy for Sjögren’s syndrome treated based on collateral disease theory. New Chin Med. (2016) 48:124–6. doi: 10.13457/j.cnki.jncm.2016.05.047

[164] Yi T . Clinical Curative Effect of Acupuncture Combined With Thunder Fire Moxibustion Treatment Fluid Deficiency of Dry Eye Syndrome. Beijing, China: Beijing University of Chinese Medicine (2014).

[165] Mardale DA Opriş-Belinski D Bojincă V Bojincă M Mazilu D Păsăran E . The physical and psychosocial impact of fatigue among patients with Sjogren's syndrome: a systematic review. J Clin Med. (2024) 13(6):1537. doi: 10.3390/jcm13061537 38541763 PMC10971331

[166] Cafaro A Arduino PG Gambino A Romagnoli E Broccoletti R . Effect of laser acupuncture on salivary flow rate in patients with Sjögren's syndrome. Lasers Med Sci. (2015) 30:1805–9. doi: 10.1007/s10103-014-1590-8 24820476

[167] Yawen L . A Study of Improving Effectiveness of Ear Points Thumbtack Needling Combining Qing Zao Bu Jin Decoction on Treatment of Primary Sjögren's Syndrome (Pattern of Yin Fluid Deficiency). Jinan, Shandong, China: Shandong University of Traditional Chinese Medicine (2022).

[168] Härtel U Volger E . Use and acceptance of classical natural and alternative medicine in Germany--findings of a representative population-based survey. Forsch Komplementarmed Klass Naturheilkd. (2004) 11:327–34. doi: 10.1159/000082814 15604623

[169] Kessler RC Davis RB Foster DF Van Rompay MI Walters EE Wilkey SA . Long-term trends in the use of complementary and alternative medical therapies in the United States. Ann Intern Med. (2001) 135:262–8. doi: 10.7326/0003-4819-135-4-200108210-00011 11511141

[170] Vincent C . The safety of acupuncture. Bmj. (2001) 323:467–8. doi: 10.1136/bmj.323.7311.467 11532826 PMC1121068

[171] Rampes H James R . Complications of acupuncture. Acupuncture Med. (1995) 13:26–33. doi: 10.1136/aim.13.1.26

[172] White A . The safety of acupuncture – evidence from the UK. Acupuncture Med. (2006) 24:53–7. doi: 10.1136/aim.24.Suppl.53

[173] Saraux A Pers JO Devauchelle-Pensec V . Treatment of primary Sjögren syndrome. Nat Rev Rheumatol. (2016) 12:456–71. doi: 10.1038/nrrheum.2016.100 27411907

[174] Sun F Tang Q Cheng W Xie X Li F Chen J . Characteristics and treatment responses of immune thrombocytopenia in patients with primary Sjögren syndrome. Int Immunopharmacol. (2023) 123:110716. doi: 10.1016/j.intimp.2023.110716 37506503

[175] Rathee P Chaudhary H Rathee S Rathee D Kumar V . Immunosuppressants: a review. Pharma Innovation. (2013) 1(12):90–101.

[176] Wan R Fan Y Zhao A Xing Y Huang X Zhou L . Comparison of efficacy of acupuncture-related therapy in the treatment of rheumatoid arthritis: a network meta-analysis of randomized controlled trials. Front Immunol. (2022) 13:829409. doi: 10.3389/fimmu.2022.829409 35320944 PMC8936080

[177] Fan Y Zhu C Ji Y Peng J Wang G Wan R . Comparison of efficacy of acupuncture-related therapies in treating acute gouty arthritis: a network meta-analysis of randomized controlled trials. Heliyon. (2024) 10:e28122. doi: 10.1016/j.heliyon.2024.e28122 38576580 PMC10990962

[178] Liu W Fan Y Wu Y Hou X Xue B Li P . Efficacy of acupuncture-related therapy in the treatment of knee osteoarthritis: a network meta-analysis of randomized controlled trials. J Pain Res. (2021) 14:2209–28. doi: 10.2147/jpr.S315956 34321920 PMC8302815

[179] Jingyue T Ruiguo L Shuaizhou Z . Effects of acupuncture therapy on negative emotions in patients with primary Sjögren’s syndrome complicated with anxiety and depression. J Med Forum. (2021) 42:134–7.

[180] Junhua D Zhimou Y Xiang L Ruiying Z Huadong Z . The combination of acupuncture and medicine in treating primary Sjgren's syndrome dry mouth for 55 cases. Chin Med Modern Distance Educ China. (2017) 15:40–2. doi: 10.3969/j.issn.1672-2779.2017.08.016

[181] Duo Y Yinxiao D . Observation on the clinical effect of the combination of traditional Chinese medicine and Western medicine in the treatment of dry eye of primary Sjogren syndrome. Guangming J Chin Med. (2020) 35:589–92. doi: 10.3969/j.issn.1003-8914.2020.04.049

[182] Xiong L Xiaofeng R . Clinical Observation on the Combined Treatment of Subcutaneous Needles and Traditional Chinese Medicine Fumigation for Sjögren's Syndrome-Associated Dry Eye Syndrome. Journal of Practical Traditional Chinese Medicine. (2024) 40(5):974-6. doi: 10.3969/j.issn.1004-2814.2024.5.syzyyzz202405070

[183] Jinying L Huijie D Meng S . Clinical observation of 34 cases of xerophthalmia treated by acupuncture. Shanghai J Acupuncture Moxibustion. (2009) 28:596. doi: 10.13460/j.issn.1005-0957.2009.10.019

[184] Rui C Rongqiu W . (2000). “ Clinical observation on therapeutic efficacy of acupuncture in 35 cases of Sjögren’s syndrome”, in: Proceedings of the Eighth Academic Conference, Clinical Branch of China Association of Acupuncture and Moxibustion (Tianjin, China: Clinical Branch of China Association of Acupuncture-Moxibustion, National Acupuncture Clinical Research Center).

[185] Fangxia Q . Clinical Study on the Effect of Acupuncture on Salivary Secretion in Patients With Primary Sjögren’s Syndrome. Beijing, China: Beijing University of Chinese Medicine (2016).

[186] Xinyao Z . Clinical Evaluation on the Effect of Acupuncture for Primary Sjögren Syndrome. Beijing, China: China Academy of Chinese Medical Sciences (2018).

[187] Lin HC Hsu CC Chen WS Ma CP Chang CM . Abs1105 the effectiveness of acupuncture at gb20 or gb20 plus bl2 in ocular dryness for patients with dry eye syndrome and SjÖgren's syndrome (acudes): a phase-2 single-blind randomized controlled study. Ann Rheumatic Dis. (2025) 84:2103–4. doi: 10.1016/j.ard.2025.06.1710 38826717

[188] Guanghui Q Huanru Q Yu T Yiyan X Xiao W Li S . Clinical observation of acupuncture treatment for dry eye acupoint in the treatment of Sjogren syndrome xeroma. Acta Chin Med. (2012) 27:1530–1. doi: 10.16368/j.issn.1674-8999.2012.11.042

[189] Baodong M Yong Z . (2012). “ Observation on therapeutic effects of eye acupuncture in 46 patients with Sjögren’s syndrome”, in: Proceedings of the sixteenth National Academic Conference on Rheumatology of China Association of Chinese Medicine (Huangshan, Anhui, China: China Association of Chinese Medicine).

[190] Dan Z Yan Z Yanting Y Xiaoxu L Yue Z Zheng S . Efficacy of electroacupuncture for patients with dry eye syndromes: a randomized controlled trial. J Acupuncture Tuina Sci. (2022) 20(6):489–98. doi: 10.1007/s11726-022-1350-4 30311153

[191] Yanchao Z Wei Y Zhengqiu C . Effects of acupuncture and moxibustion on tear-film of the patients with xerophthalmia. J Traditional Chin Med. (2007) 27(1):258–60. 18246680

[192] Jing-wen L Yao-dong Z Ling Z Cong H . Efficacy observation of acupuncture for dry eye syndrome of lung-yin deficiency pattern. J Acupuncture Tuina Sci. (2021) 19(19):72–7. doi: 10.1007/s11726-021-1228-x 30311153

[193] wei L Bin L Yanli Z . (2004). “ Observation on therapeutic effects of acupuncture for 60 patients with Sjögren’s syndrome”, in: Collection of Papers of the First International Academic Conference on Integrated Chinese and Western Medicine for Rheumatism (Harbin, Heilongjiang, China: Rheumatology Committee of Chinese Society of Integrated Traditional Chinese and Western Medicine).

[194] Geshu D . Clinical observation of 40 patients with Sjögren’s syndrome treated by acupuncture. Guiding J Traditional Chin Med Pharm. (2010) 16:61–2. doi: 10.3969/j.issn.1672-951X.2010.10.033

[195] Bai H Yu P Yu M . Effect of electroacununcture on sex hormone levels in patients with Sjögren's syndrome. Zhen Ci Yan Jiu. (2007) 32:203–6. doi: 10.3969/j.issn.1000-0607.2007.03.015 17691581

[196] Yaju P Guiqin J Jinling F Zhisheng C Xin W . Effects of acupuncture on tear secretion in patients with Sjögren’s syndrome. China J Chin Ophthalmol. (2003) 13(1):21–3. doi: 10.3969/j.issn.1002-4379.2003.01.006

[197] Yang G Huayu M Zheng L . Clinical observation of Sjögren’s syndrome treated by acupuncture combined with intraductal dexamethasone injection of salivary glands. J Med Inf. (2009) 22:1906. doi: 10.3969/j.issn.1006-1959.2009.09.129

[198] Shixin D Chenggang L Xiao W . Observation on the therapeutic effect of acupuncture at yanglao acupoint(si6)for dry eye syndrome associated with primary Sjögren’s syndrome. Shandong J Traditional Chin Med. (2026) 45:277–82. doi: 10.16295/j.cnki.0257-358x.2026.03.010

[199] Xiulian Z . Analysis of clinical efficacy of yiguan decoction combined with acupuncture and moxibustion in treating primary Sjögren’s syndrome. Chin Sci Technol J Database (Full-text Edition): Med Health. (2021) 2021(9):029–031.

[200] Jiyou K Haixia F Yan W . Acupuncture combined with warm Fu Ge decoction for dry syndrome of yang deficiency type. Jilin J Chin Med. (2016) 36(9):948–50. doi: 10.13463/j.cnki.jlzyy.2016.09.024

[201] Jinhuan Z Jianyong Z Jingjing X . Clinical observations on combined use of acupuncture and medicine for primary Sjogren syndrome. Shanghai J Acupuncture Moxibustion. (2018) 37(12):1399–404. doi: 10.13460/j.issn.1005-0957.2018.12.1399

[202] Jiayi F . Clinical Observations on Combined Use of Acupuncture and Medicine for the Primary Sjögren's Syndrome of Kidney Essence Deficit. Jinan, Shandong, China: Shandong University of Traditional Chinese Medicine (2024).

[203] Xiaoya Y Yi W . Clinical observation on 42 cases of Sjögren’s syndrome treated with combination of acupuncture and medicine. J Shandong Univ Traditional Chin Med. (2005) 29(4):292–309. doi: 10.3969/j.issn.1007-659X.2005.04.019

[204] Zi L . Clinical Study on the Application of Tongyuan Therapy on Primary Sicca Syndrome. Guangzhou, Guangdong, China: Guangzhou University of Chinese Medicine (2018).

[205] Yan C Huimin L Xinfang T . Clinical observation on the therapeutic effect of acupuncture combined with traditional Chinese medicine in treating primary Sjögren’s syndrome. New Mom New Born. (2021) 2021(2):46.

[206] Junhua G Hua W . (2016). “ Clinical observation on treating Sjögren’s syndrome from the liver by combined acupuncture and Chinese herbal medicine”, in: Proceedings of the Inaugural Conference of the External Therapy Techniques Professional Committee, World Federation of Chinese Medicine Societies & the 1st International Academic Conference on External Therapy Techniques (Ji Nan, China: World Federation of Chinese Medicine Societies), 139–42.

[207] Qing G Shiping Q Baocheng L . Linical observation on the efficacy of combined acupuncture and herbal medicine for Sjögren’s syndrome. J Li-Shizhen Traditional Chin Med. (2008) 19:195–6. doi: 10.3969/j.issn.1008-0805.2008.01.111

[208] Lanping H Youwen C . Clinical observation on clinical effects of acupuncture combined with Chinese herbal medicine in the treatment of primary Sjögren’s syndrome. Shenzhen J Integrated Traditional Chin Western Med. (2018) 28:73–5. doi: 10.16458/j.cnki.1007-0893.2018.05.033

[209] Baoguo Z . Efficacy of eye acupuncture, Chaihu Guizhi Ganjiang decoction combined with western medicine in the treatment of Sjögren syndrome. World J Complex Med. (2024) 10:21–4. doi: 10.11966/j.issn.2095-994X.2024.10.07.06

[210] Yanjuan O Yanyan G Jianjun Z . Clinical research on treating Sjogren's syndrome by acupuncture combined with medicine. Clin J Chin Med. (2013) 5:48–9. doi: 10.3969/j.issn.1674-7860.2013.03.026

[211] Huiqiang W . The Clinical Study of Ziyin Runzao Decoction Combined With Acupuncture in the Treatment of Primary Sjogren’s Syndrome With Deficiency of Qi-Yin. Harbin, Heilongjiang, China: Heilongjiang University of Chinese Medicine (2022).

[212] Xuefei C . Clinical Observation of Acupuncture and Medicine Combined Treatment of Primary Sjögren's Syndrome With Yin Deficiency and Jin Deficiency. Tianjin, China: Tianjin University of Traditional Chinese Medicine (2021).

[213] Lin M Kunying L Xiumin Z Fengkuan C Qiang C Hong L . Clinical observation on 35 cases of Sjögren’s syndrome treated by acupoint injection combined with acupuncture. Chin Acupuncture Moxibustion. (2004) 24(9):37–8. doi: 10.13703/j.0255-2930.2004.09.019

[214] Xiaowen S . Clinical observation on 38 cases of ocular-oral Sjögren’s syndrome treated with oral Chinese herbal medicine combined with auricular point pressing. Jiangsu J Traditional Chin Med. (2005) 26(10):39–40. doi: 10.3969/j.issn.1672-397X.2005.10.023

